# Bcl-xL activity influences outcome of the mitotic arrest

**DOI:** 10.3389/fphar.2022.933112

**Published:** 2022-09-15

**Authors:** M. Suleimenov, S. Bekbayev, M. Ten, N. Suleimenova, M. Tlegenova, A. Nurmagambetova, S. Kauanova, I. Vorobjev

**Affiliations:** ^1^ Department of Biology, School of Sciences and Humanities, Nazarbayev University, Nur-Sultan, Kazakhstan; ^2^ National Laboratory Astana, Nazarbayev University, Nur-Sultan, Kazakhstan; ^3^ School of Engineering and Digital Science, Nazarbayev University, Nur-Sultan, Kazakhstan

**Keywords:** mitotic arrest, anti-mitotic drugs, apoptosis, Bcl-2 proteins, flow cytometry, live cell imaging

## Abstract

Microtubule-targeting (MT) drugs taxanes and vinca alkaloids are widely used as chemotherapeutic agents against different tumors for more than 30 years because of their ability to block mitotic progression by disrupting the mitotic spindle and activating the spindle assembly checkpoint (SAC) for a prolonged period of time. However, responses to mitotic arrest are different—some cells die during mitotic arrest, whereas others undergo mitotic slippage and survive becoming able for proliferation. Using normal fibroblasts and several cancer cell types we determined two critical doses, T1 and T2, of mitotic inhibitors (nocodazole, Taxol, and vinorelbine). T1 is the maximal dose cells can tolerate undergoing normal division, and T2 is the minimal mitostatic dose, wherein > 90% of mitotic cells are arrested in mitosis. In all studied cell lines after treatment with mitotic inhibitors in a dose above T2 cells had entered mitosis either die or undergo mitotic slippage. We show that for all three drugs used cell death during mitotic arrest and after slippage proceeded *via* mitochondria-dependent apoptosis. We determined two types of cancer cells: sensitive to mitotic arrest, that is, undergoing death in mitosis (DiM) frequently, and resistant to mitotic arrest, that is, undergoing mitotic slippage followed by prolonged survival. We then determined that inhibition of Bcl-xL, but not other anti-apoptotic proteins of the Bcl-2 group that regulate MOMP, make resistant cells susceptible to DiM induced by mitotic inhibitors. Combined treatment with MT drugs and highly specific Bcl-xL inhibitors A-1155643 or A-1331852 allows achieving 100% DiM in a time significantly shorter than maximal duration of mitotic arrest in all types of cultured cells tested. We further examined efficacy of sequential treatment of cultured cells using mitotic inhibitors followed by inhibitors of Bcl-xL anti-apoptotic protein and for the first time show that sensitivity to Bcl-xL inhibitors rapidly declines after mitotic slippage. Thus sequential use of mitotic inhibitors and inhibitors of Bcl-xL anti-apoptotic protein will be efficient only if the Bcl-xL inhibitor will be added before mitotic slippage occurs or soon afterward. The combined treatment proposed might be an efficient approach to anti-cancer therapy.

## Introduction

Antimitotic drugs, such as paclitaxel (Taxol) and vinorelbine are widely used for treatment of numerous solid tumors including breast cancer, prostate cancer, and others. These drugs directly interact with microtubules stabilizing (taxanes) or destabilizing (vinca alkaloids) them and at concentrations ≥ 100 nM always induce mitotic arrest in many cell lines through inhibition of dynamic instability of microtubules and continuous activation of the spindle assembly checkpoint (SAC) (Jordan et al., 1992; Jordan et al., 1996; Ikui et al., 2005; Musacchio and Salmon, 2007; Gascoigne and Taylor, 2008; Riffell et al., 2009).

After mitotic arrest cells that slip into interphase can die, remain alive and undergo senescence, or start proliferating (Rieder and Maiato, 2004; Ikui et al., 2005; Blagosklonny, 2007; Shi et al., 2011).

Although mitotic arrest induced by MT inhibitors is universal in all types of proliferating cells, the outcome of the arrest (cell death or prolonged survival) differs significantly among cancer and normal cell lines (Gascoigne and Taylor, 2008; Shi et al., 2008; Ghelli Luserna di Rorà et al., 2019).

The most straightforward way to enhance the efficacy of anti-microtubule drugs is to combine them with specific agents stimulating apoptosis specifically during mitotic arrest (Shi et al., 2011; Barille-Nion et al., 2012; Haschka et al., 2015). The key feature of such treatment is that the drug used for killing mitotic cells would not affect cells at other stages of the cell cycle. One of the most efficient drugs is navitoclax (ABT-263) that inhibits several anti-apoptotic Bcl-2 proteins (Vogler et al., 2009). Navitoclax was shown to induce dramatic decrease in cell survival under the action of Taxol and K5I (kinesin 5 inhibitor) and suggested to be a promising anti-cancer agent (Shi et al., 2011).

Despite of promising results obtained during clinical trials and increased effectiveness against mitotically arrested cells during combined treatment with navitoclax and Taxol (Mohamad Anuar et al., 2020). Navitoclax was shown to cause significant side-effects in many patients, that is, severe neutropenia that is explained by its activity against Bcl-2 protein responsible for blood cell differentiation and survival (Puglisi et al., 2011; Chen et al., 2017). Then the new highly selective Bcl-xL inhibitors were developed (Tao et al., 2014; Leverson et al., 2015). These drugs (A-1155643 and its orally bioavailable analog A-1331852) showed promising results in the inhibition of renal carcinomas (Grubb et al., 2021) and non-small-cell lung cancer (NSCLC) cells (Li et al., 2022). However, results of the use of these inhibitors together with Taxol (Leverson et al., 2015; Faqar-Uz-Zaman et al., 2018) are incomplete and direct comparison with navitoclax/Taxol treatment is lacking. The question about sequential use of mitotic inhibitors followed by Bcl-xL inhibitors also remains open.

Taking into account large variability of responses of cancer cell lines on the mitotic inhibitors, the question is whether duration of mitotic arrest and its outcome depends on the drug used? The existing data are controversial (Gascoigne and Taylor, 2008; Shi et al., 2008; Shi et al., 2011; Tsuda et al., 2017). Particularly, comparison of different inhibitors of microtubule dynamic instability such as taxanes, vinca alkaloids, and nocodazole is incomplete. Answer is very important since it might affect therapeutic programs.

Second question is about the relative role of different anti-apoptotic proteins in control of death in mitosis (DiM) during prolonged mitotic arrest. Role of anti-apoptotic proteins in the outcome of mitotic arrest was elucidated mainly using the siRNA approach (Shi et al., 2011; Lee et al., 2014; Hüsemann et al., 2020) that is not convenient to be used for animal models and clinical trials as a part of combinatorial treatment. This analysis requires, first, to determine cell culture that is prone to mitotic slippage under mitotic arrest induced by different drugs and then evaluate its behavior under the treatments with different highly specific inhibitors of Bcl-2 proteins.

Third question is about the minimal duration of mitotic arrest when DiM could be stimulated by an additional treatment with Bcl-2 inhibitors and whether these inhibitors can stimulate death after mitotic slippage.

To understand how cell response after treatment with MT drugs is regulated by anti-apoptotic Bcl-2 proteins and duration of mitotic arrest, we used prolonged live-cell imaging technique to track individual cell fates and flow cytometry to conduct population analyses. We determined two types of cancer cells: sensitive to mitotic arrest induced by anti-mitotic drugs, that is, undergoing DiM frequently, and resistant to mitotic arrest, that is, undergoing slippage from mitotic into interphase state. We next confirmed that cell death during or after mitotic arrest induced by anti-mitotic drugs always proceeds *via* the apoptosis pathway starting with mitochondrial outer membrane permeabilization (MOMP) and is followed by activation of caspases 3/7. Inhibition of caspases in DiM sensitive cells did not prevent MOMP but allowed some cells to escape mitotic arrest. Inhibition of Bcl-xL in the slippage-prone cells by low doses of specific Bcl-xL inhibitors A-1155643 or A-1331852 (Tao et al., 2014) made them DiM sensitive. Sequential treatment of resistant cells with mitotic inhibitors followed by inhibition of Bcl-xL with A-1155643 or A-1331852 also resulted in a rapid cell death of slippage-prone cell lines if they were still in mitotic stage or escape mitosis shortly before addition of the Bcl-xL inhibitor. This data might be beneficial for further development of anti-cancer therapy using taxane- or vinorelbine-based programs.

## Materials and methods

### Reagents and antibodies

Microtubule inhibitors nocodazole (Cat. #M1404), Taxol (Cat. # PHL89806), vinorelbine (Cat. #V2264), broad spectrum protein kinase inhibitor staurosporine (Cat. # 19-123), tetramethylrhodamine ethyl ester (TMRE, Cat. #87917), and propidium iodide (PI, Cat. #P4170) were purchased from Sigma-Aldrich. CellEvent™ Caspase 3/7 Green Detection Reagent (Cat. #C10423), Hoechst 33342 (Cat. # 62249), and RNase A (Cat. # EN0531) were purchased from Thermo Fisher Scientific. Venetoclax (Cat. #S8048), S63845 (Cat. #S8383), navitoclax (Cat. #S1001), A-1155463 (Cat. #S7800), and barasertib (AZD1152-HQPA, Cat. #S1147) were purchased from Selleckchem Inc. Z-VAD(OH)-FMK (Cat. # ab120382), primary antibodies against Bcl-xL (Cat. # ab32370) and Bcl-2 (Cat. # ab182858), and horseradish peroxidase–conjugated goat anti-rabbit secondary antibodies (Cat. # ab6721) were purchased from Abcam (United States).

### Cell lines

In total four cancer cell lines (HeLa, U-118, A549, and PC-3), two minimally transformed cell lines (HaCaT and NIH-3T3), and one normal cell line (primary human fibroblasts or PHF kindly provided by B. Soltankulov) were maintained according to a regular recommendation of ATCC for mammalian cultures in Dulbecco’s modified Eagle medium (Thermo Fisher Scientific, Cat. # 11965092) or DMEM/F-12 (Thermo Fisher Scientific Cat. # 21331020) supplemented with 10% fetal bovine serum (Thermo Fisher Scientific, Cat. 10100147), 4–8 mM of l-glutamine (Sigma, Cat. # G7513), and antibiotics penicillin–streptomycin (Sigma-Aldrich, Cat. # P4333).

### Microscopic image acquisition and analysis

All microscopic observations were performed on an automated Zeiss Cell Observer microscope (Carl Zeiss GmbH), equipped with a heating incubator chamber and using ZEN software. Images were recorded using ORCA-Flash4.0 V2 (Hamamatsu Photonics, Hamamatsu, Japan).

Bright-field and fluorescent time-lapse microscopy were used to record life histories of individual cells. Prior to image acquisition, the cells were seeded into 48-well plates (TPP, Cat. # 92448) or glass-bottom 8-well chambers (Ibidi, Cat. 80,826) and incubated overnight. Then culture media was changed to CO_2_-independent medium (Thermo Fisher Scientific, Cat. # 18045088) with 10% FBS and 4–8 mM l-glutamine (Sigma, Cat. #G7513) that already contained a working concentration of a drug. Duration of the observation was 48–72 h with a 15-min interval between the frames under an objective Plan–Apochromat ×10/0.45.

For fluorescence microscopy Plan–Apochromat objectives ×20/0.8 (dry) and ×63/1.46 (oil immersion), LED light source Colibri 2 and the following filter cubes were used: DAPI (excitation filter 335–383 nm, a beam splitter 395 nm, and emission 420–470 nm), GFP (excitation filter 450–490 nm, a beam splitter 495 nm, and emission 500–550 nm), and DsRED (excitation filter 538–562 nm, a beam splitter 570 nm, and emission 570–640 nm).

Acquired time-lapse series were first converted from *.czi (ZEN software) into TIFF format. Then time-lapse series were manually analyzed in Fiji software using the cell counter plugin. Data on individual cells was collected in Excel files and then used to build representative figures in GraphPad Prism 8 software.

Density of cells at the beginning of the time-lapse observation was in the range of 40%–50% for experiments on cell proliferation and up to 90% for staurosporine treatment. Since the duration of time-lapse observations was 48–72 h, cells from the control group (non-treated) were seeded at a lower density (20–30% confluency), whereas at mitostatic concentrations of the drugs the cells were seeded at a higher density to ensure that enough mitotic cells will be present in the field of view for analysis.

### Flow cytometry

DNA staining with propidium iodide was performed as described previously (Potashnikova et al., 2019). In brief, cells were seeded into 12-well cell culture plates, then after 24–48 h MT inhibitors (3–1000 nM) were added to fresh culture medium, and cells were further incubated for 48 h in 37°C incubator, for control cells fresh culture medium containing equivalent amount of DMSO was added. After incubation cells were harvested by standard trypsinization procedure and washed with 500 ul of PBS at room temperature by centrifugation. Cell pellet was then fixed with 70% ice-cold ethanol and kept at 4°C for > 4 h. Cells were pelleted again by centrifugation and stained with 30 ug/ml of PI and 5 ug/ml of RNase A.

Cells were analyzed using an Attune NxT flow cytometer (Thermo Fisher Scientific) at Ex.488 nm/Em.550–630 nm for PI. The results obtained from flow cytometry were analyzed using Flow Jo software (BD, Ashland, OR). All measurements were performed at least in triplicate. Over 30 000 events were analyzed in each tube.

### Western blotting

#### Cell preparation

Cells for immunoblotting were grown in a T-25 flask until the 90%–100% confluency was reached. Untreated cells were collected using a standard protocol for harvesting.

Before incubation in Taxol, the cells were grown in a T-25 flask until the 90%–100% confluency was reached. Then the old medium was changed to a new culture medium (6 ml) containing 1 µM of Taxol. After 12 or 24 h of incubation in Taxol, mitotic cells were gently shaken and collected by centrifugation. Purity of the fraction of mitotic cells was confirmed by flow cytometry showing absence of G1 and sub-G1 cells (data not shown).

#### Immunoblotting

Cells (35 × $10^{4}$ ) were washed with 1X PBS and resuspended in 1X Laemmli buffer (1% SDS, 10% glycerol, and 0.05M Tris pH6.8). After, cells were scraped and transferred to a microcentrifuge tube. To complete lysis, cells were sonicated for 15s and heated at 95°C for 5 min. Then the lysates were centrifuged for 2 min at 10,000 rpm. For protein quantification, a colorimetric Bradford assay kit (ab102535, Abcam, United States) was used. The total protein was separated using 12% sodium dodecyl sulfate–polyacrylamide (SDS-PAGE) gel (120V, 1.5 h) and electro-transferred on PVDF (IPFL00010, Immobilon-FL, Millipore Corporation, Ireland) membranes (20V, overnight). The membranes were treated with blocking reagents (-5% non-fat dry milk in 1X TBST (Tris-buffered saline containing 0.1% Tween 20) or 5% BSA (A7030, Sigma-Aldrich, United States) in 1X TBST for pSer62 Bcl-xL) for 1 h at room temperature. Following three 5 min washes with 1X TBST, membranes were incubated with primary antibodies in 3% BSA at room temperature for the next 1 h. Protein lysates were incubated with the following primary antibodies with the respective dilutions: Bcl-xL (A95103, 1:500, antibodies, Cambridge, UK), Bcl2 (ab182858, 1:2000, Abcam, United States), Mcl-1 (ab32087, 1:500, Abcam, United States), pSer62 Bcl-xL (A94215, 1:500, antibodies, Cambridge, UK), and α-Tubulin (#2144S, 1:1000, Cell Signaling, The Netherlands). After extensive washes, incubation with HRP-conjugated anti-rabbit secondary antibodies was conducted for 1 h at room temperature. As the secondary antibody, goat anti-rabbit IgG H&L HRP (ab6721, 1:5000, Abcam, United States) was used. Immunoblots were visualized with an ECL reagent (ab65623, Abcam, United States) using a Bio-Rad imaging system.

### Statistical analysis

For comparison of two mean values, Student’s t-test was used. For comparison of multiple mean values, Tukey’s ordinary one-way ANOVA or ordinary one-way ANOVA with Dunnet’s multiple comparison test was used. *p* values below 0.05 were considered significant. All statistical analyses were performed in GraphPad Prism 8 software (GraphPad Software Inc.). All tables were constructed in Microsoft Excel software.

## Results

### Flow cytometry and time-lapse microscopy reveal similar dose-dependent response to MT drugs in different cell lines

Taxol and vinorelbine induces mitotic arrest usually at concentrations above 10–20 nM, and cells undergo aberrant mitosis at lower concentrations of these drugs (Masuda et al., 2003; Ikui et al., 2005). At concentrations in the range of tens of nanomoles (10–100 nM) cancer cells show large inter- and intraline variation (Gascoigne and Taylor, 2008; Shi et al., 2008, 2011). Our previous cytometric analysis of lymphoid cell lines showed that exposure to different doses of Taxol, nocodazole, and vinorelbine for 24 h leads to accumulation of cultured cells in G2/M in a non-linear fashion with minimal efficient dose of each drug in the range 30–100 nM (Potashnikova et al., 2019). This prompted us to analyze variation in response to mitotic inhibitors in a dose-dependent way in more details. Micromolar doses of mitotic inhibitors are always sufficient for significant disruption of the mitotic spindle, whereas nanomole doses usually have no effect (Shi and Mitchison, 2017). For data uniformity, we used the range of concentrations from 1 nM (or 3 nM in some cases) to 1000 nM.

To properly describe the variation in cell fates as a dose-dependent response, we first determined minimal effective doses of 3 MT drugs (nocodazole, Taxol, and vinorelbine) for seven cell lines (HaCaT, U-118, PHF, HeLa, HaCaT, A549, and PC-3) using flow cytometry and time-lapse microscopy.

**FIGURE 1:**
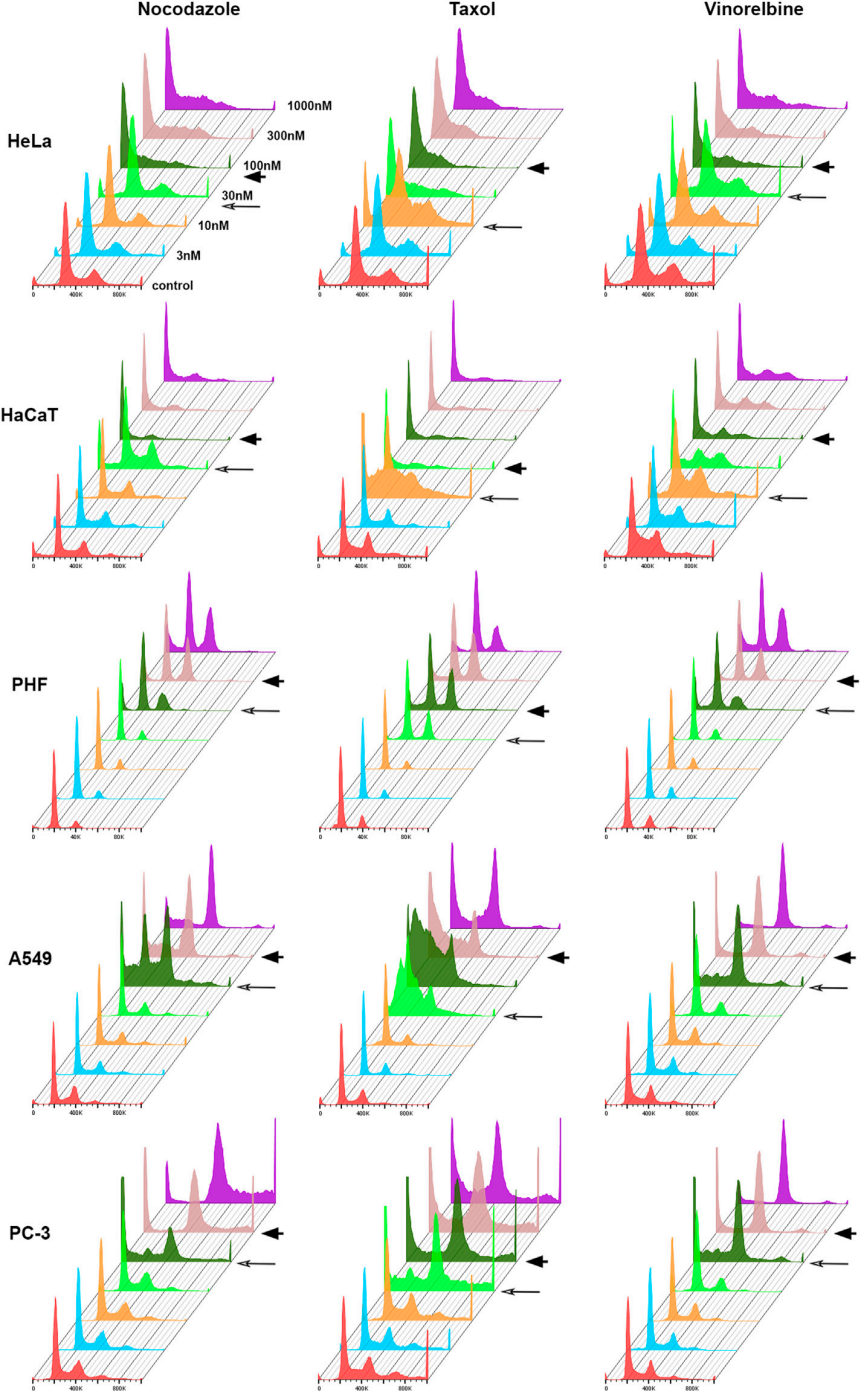
Histograms showing cell distribution according to DNA content after 48 h treatment with anti-microtubule drugs. Minimal threshold concentrations (T1) for each drug are indicated by long arrow, and maximal threshold concentrations (T2)—by arrowhead.

For each cell line–drug type pair, we determined two threshold doses (minimal and maximal) that separated normal cell cycle distribution at lower doses from significant G2/M or sub-G1 accumulation in higher doses of MT drugs (Figure 1). Above the maximal threshold dose, distribution of cells in the cell cycle remained highly conservative within the cell line. HeLa and HaCaT cells accumulated mainly in sub-G1 population, whereas A549 and PC-3 accumulated mainly in G2/M (Figure 1). Based on the cell response in dose above the maximal threshold, we classified our cultures as sensitive to mitotic arrest (HeLa and HaCaT) or resistant (A549 and PC-3). Then for the more detailed analysis of cell response to different doses of 3 MT drugs, we evaluated behavior of our cell lines using time-lapse microscopy.

**FIGURE 2:**
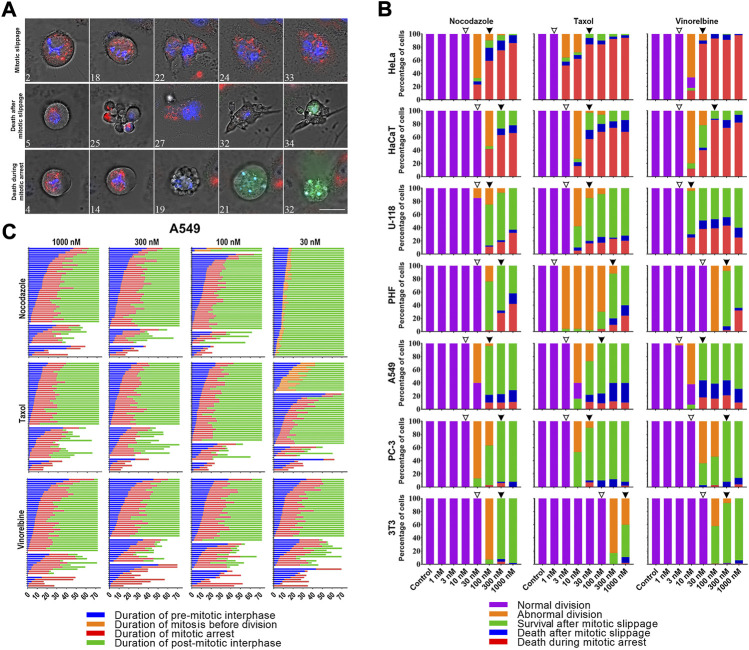
**(A)** Time-lapse sequences illustrating three fates (mitotic slippage, death after mitotic slippage, and death in mitosis) exhibited by A549 cells under the treatment with 1 uM Taxol. Numbers indicate time from the moment of mitotic entry in hours. Mitochondrial membrane potential was visualized after staining with 50 nM TMRE (red), active caspases 3/7 with caspases 3/7 detecting reagent (green), and nuclei with 200 nM Hoechst 33342(blue). The images were acquired using a Planapo ×63 oil immersion objective with NA = 1.46. Scale bar = 20 µm. **(B)** Threshold-dependent response of HeLa, HaCaT, U-118, PHF, A549, PC-3, and 3T3 cell lines after 72 h of treatment with 1–1000 nM doses of nocodazole, Taxol, and vinorelbine. From top to bottom cell lines are placed in order of decreasing sensitivity to death in mitosis. Threshold doses are indicated by empty (T1) and filled (T2) arrowheads. **(C)** Life histories of individual A549 cells treated with 30–1000 nM doses of nocodazole, Taxol, or vinorelbine. Each horizontal line represents a single cell. Numbers along the x-axis indicate the time from the moment addition of the drug in hours.

Depending on the drugs’ dose, for all cell lines mitosis always resulted in one of the following (Gascoigne and Taylor, 2008): (i) normal division, (ii) several types of abnormal division, or (iii) prolonged mitotic arrest followed by either death during mitotic arrest (DiM) or mitotic slippage (Figure 2A). Abnormal divisions include unequal division, death of one or both daughter cells after division, division after mitotic arrest (when duration of mitosis was > 3 times longer than average duration of mitosis in a respective control group), and division followed by cell cycle arrest. After mitotic slippage, the cells either died or survived until the end of the experiment.

For each cell line–drug type pair, we observed similar threshold-dependent response as in flow cytometry experiments. A detailed time-lapse microscopic analysis confirmed the presence of two critical doses (Figure 2B). The first threshold dose (T1) is defined as the maximal dose cells can tolerate undergoing normal division in > 90% of cases, and the second threshold dose (T2) is the minimal mitostatic dose, wherein > 90% of mitotic cells are arrested in mitosis. In T1 and lower doses, the cells divided normally and duration of mitosis does not change (Supplementary Table S1). In between T1 and T2 many cells divided abnormally or were arrested in mitosis. In T2 and higher doses, all mitotic cells were arrested in mitosis for several hours followed by either death during mitosis or mitotic slippage.

For all cell lines, except 3T3 cells, T1 doses were in the range of 10–30 nM for nocodazole, in the range of 1–3 nM for Taxol and 3–10 nM for vinorelbine. T2 doses were similar for all cultures and drugs tested except high sensitivity of HeLa cells to Taxol (Supplementary Table S1).

Relative frequencies of three cell fates in T2 and higher doses varied greatly among the cell lines studied (interline variation is shown in Figure 2B). During the microscopic analysis of the inter-line variation, the most sensitive lines with the highest frequency of DiM were HeLa (80–95% DiM) and HaCaT (70–90% DiM). Resistant cell lines with high frequency of mitotic slippage were U-118 (60–80%), PHF (60–80%), A549 (80–90%) PC-3 (∼95%), and 3T3 (99%). U-118, PHF, and A549 cells demonstrated relatively high frequency of death after slippage, whereas PC-3 and 3T3 cells always survived after slippage till the end of experiment (Supplementary Figure S1). Distribution of cell fates within each cell line (intra-line variation) remained highly conservative for all doses above the T2 level of all three drugs (Figures 2B,C; Supplementary Figure S1). To assess dose-dependent intra-line variation in detail, we analyzed A549 and U-118 cells, which showed all three fates following mitotic arrest at relatively high frequencies. Our results for A549 and U-118 cell lines explicitly show that even 30-fold (from 30 to 1000 nM) and 100-fold (from 10 to 1000 nM) increase in the dose of vinorelbine, respectively, did not significantly change relative frequencies of cell fates (Figure 2B).

Duration of mitotic arrest varied among different cell lines but remained conservative within each cell line for all doses above T2 level of all three drugs. For each cell culture mean duration of mitotic arrest in response to three drugs differed not more than 33% (Supplementary Figure S2; Supplementary Table S1-S4).

### Cell death during mitotic arrest and after mitotic slippage proceeds *via* MOMP and activation of caspases 3/7

Cell death after treatment with MT drugs has been reported to proceed caspase-dependent (Gascoigne and Taylor, 2008; Shi et al., 2011) or caspase-independent (Niikura et al., 2007) apoptosis, but detailed evidence at the individual cell level is lacking. We studied progression of apoptosis initiated by mitotic arrest in a detailed manner by tracking MOMP and caspases 3/7 activity (Vorobjev and Barteneva, 2015) in DiM sensitive HeLa and slippage prone A549 cells after treatment with 1 µM Taxol. As a positive control for apoptosis, we treated our cells with 1 µM staurosporine.

**FIGURE 3:**
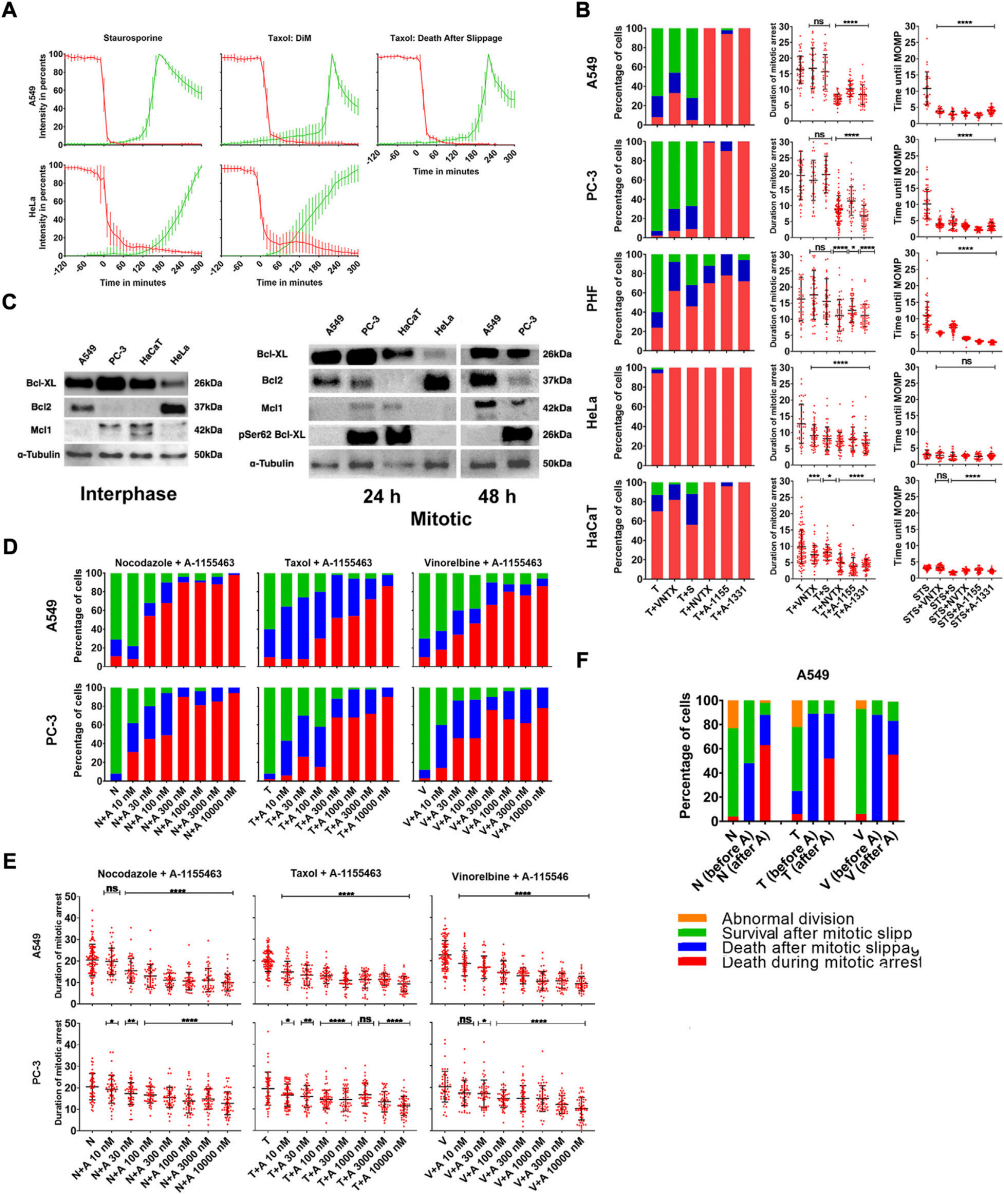
**(A)** Averaged and normalized intensity values from TMRE (red) and caspases 3/7 detecting reagent (green) for slippage prone A549- and DiM-sensitive HeLa cells after treatment with 1 µM staurosporine (positive control for apoptosis) or 1 µM Taxol. Vertical bars represent SD. N = 10–15 cells for each category. **(B)** Distribution of fates and average durations of mitotic arrest (in hours) of PHF, slippage prone A549 and PC-3, and DiM-sensitive HeLa and HaCaT cells in response to the treatment with Taxol (T) in combination with Bcl-2 inhibitor venetoclax, Mcl-1 inhibitor S63845 (S), Bcl-2, Blc-xL, and Bcl-w inhibitor navitoclax (NVTX) or specific Bcl-xL inhibitors A-1155463 (A-1155) and A-1331852 (A-1331). As a positive control, five cell lines were also treated with staurosporine (STS) in combination with each inhibitor of anti-apoptotic Bcl-2 proteins. The y-axis shows time from the moment of STS addition until MOMP in hours. **(C)** Immunoblotting showing expression of Bcl-2, Bcl-xL, phosphorylated Bcl-xL and Mcl-1 in slippage-prone (A549, PC-3) and DiM sensitive (HeLa, HaCaT) cell lines in the interphase and during mitotic arrest. **(D)** Distribution of fates and **(E)** average durations of mitotic arrest (in hours) of A549 and PC-3 cell lines after the treatment with 1 µM doses of Nocodazole (N), Taxol (T) or Vinorelbine (V) in combination with 10-10000 nM doses of A-1155463 **(A)**. **(F)** Response of slippage prone A549 cells to the sequential treatment with MT drugs and A-1155453. The cells were incubated in MT drugs Nocodazole (N), Taxol (T) or Vinorelbine (V) for 24 h and 4 h after removal of MT drugs, 1 µM A-1155463 **(A)** was added. For each MT drug, first column represents overall cell response after removal of the drug (N, T, or V) when no inhibitor of Bcl-xL was added. Second column represents response of cells that underwent mitotic slippage before addition of A-1155463 (N, T, or V (before A)). Third column represents response of cells that remained in mitotic state at the moment of A-1155463 addition (N, T or V (after A)).

In both cell lines, nearly all cell deaths after treatment with Taxol or staurosporine started with MOMP along with cell shrinkage and were followed by activation of caspases 3/7. Dynamics of MOMP was always rather rapid—90% drop of membrane potential happened within 20 min, whereas caspases 3/7 activation varied depending on the cell line (Figure 3A) but within cell line was the same for each drug (data not shown).

After treatment with staurosporine, cells from both cell lines started to shrink soon after addition of the drug but retained their mitochondrial membrane potential for several hours. After treatment with Taxol, the cells were arrested in mitosis and retained their mitochondrial membrane potential for the whole duration of mitotic arrest in case of DiM and for the whole duration of mitotic arrest and certain time after slippage in case of death after slippage.

Fluorescence intensity from TMRE remained constant and was maximal/nearly maximal before initiation of MOMP when it rapidly drops down in individual cells. MOMP was defined as 90% loss in fluorescence intensity from TMRE. The moment of activation of caspases was recognized at 10% of maximal fluorescence intensity from caspases 3/7 detecting reagent.

In A549 cells treated with staurosporine, MOMP occurred 11 h after addition of the drug. In A549 cells treated with Taxol, MOMP occurred 19 h after mitotic arrest in case of DiM and within 24 h of post-slippage interphase in case of death after slippage. In HeLa cells treated with staurosporine, MOMP occurred 4 h after addition of the drug (Figure 3B). In HeLa cells treated with Taxol, MOMP occurred 10 h after being arrested in mitosis in case of DiM (Figure 3B). It is important to notice that mitotic cells undergoing MOMP were not able to slip from mitotic state into the interphase and caspase activation always happened during mitotic state, if MOMP had taken place. On the other hand, cells undergoing death after slippage always started with MOMP after slippage, that is, in the interphase state.

### Inhibition of Bcl-xL, but not other anti-apoptotic Bcl-2 proteins, sensitizes slippage-prone cells to apoptosis during mitotic arrest

Confirming that mitotic arrest initiates cell death always through apoptotic execution, we further analyzed role of anti-apoptotic proteins in this process using highly specific inhibitors for individual Bcl-2 group proteins. When used alone, each Bcl-2 inhibitor had no visible effect on cells’ survival and proliferation (data not shown). Only PC-3 cells showed moderate sensitivity, with ∼20% of the interphase cells dying under the treatment with A-1155463, A-1331852, or navitoclax.

Anti-apoptotic Bcl-2 proteins may play important role in the regulation of apoptosis during mitotic arrest. Certain evidence confirms that resistance to DiM depends on expression of Mcl-1 and Bcl-xl, and DiM sensitive cells usually express Bcl-xL at low levels, whereas Mcl-1 degrades rapidly during mitotic arrest (Shi et al., 2011; Sloss et al., 2016).

Expression of three major anti-apoptotic proteins shows large degree of variation both in control and after collection of mitotic cells after arrest with Taxol (Figure 3C and Supplementary Figure S4). Assessing the role of individual anti-apoptotic Bcl-2 members in the regulation of apoptosis during mitotic arrest in more details, we treated resistant PC-3, A549, and PHF cells as a reference and sensitive HeLa and HaCaT cells with Taxol in combination with 10 µM S63485 (Mcl-1 inhibitor), 10 µM venetoclax (Bcl-2 inhibitor), 10 µM A-1155463 (Bcl-xL inhibitor), 10 uM A-1331852 (Bcl-xL inhibitor), or 10 µM navitoclax (Bcl-2/Bcl-xL/Bcl-w inhibitor).

Inhibition of each member of anti-apoptotic Bcl-2 proteins sensitized PHF cells to DiM. Time until DiM shortened significantly in the presence of Bcl-xL inhibitors (A-1551463, A-1331852, or navitoclax), but not in the presence of venetoclax or S63845 (Figure 3B; Supplementary Table S5, S6). We conclude that normal fibroblasts are sensitized to DiM by inhibiting any anti-apoptotic protein of the Bcl-2 group with inhibitors of Bcl-xL showing the strongest effect.

Inhibition of Bcl-xL during mitotic arrest sensitized all resistant cells to apoptosis and complete inhibition accelerated DiM. When slippage prone A549 and PC-3 cells were treated with Taxol in combination with A-1155463, A-1331852, or navitoclax, > 90% of mitotically arrested cells died in mitosis (Figure 3B). For both cell lines average time spent in mitosis was twice shorter under Taxol + A-1155463, A-1331852, or navitoclax in comparison with Taxol only (Figure 3B; Supplementary Table S5, S6). However, inhibition of Bcl-2 and Mcl-1 did not sensitize A549 and PC-3 cells to DiM. Under treatment with Taxol + Venetoclax or S63485 most cells underwent slippage and survived until the end of the observation. Duration of mitotic arrest in the presence of venetoclax or S63845 for both cell lines changed insignificantly and was similar to the duration of mitotic arrest under the action of Taxol only (Figure 3B; Supplementary Table S5, S6).

Inhibition of each anti-apoptotic Bcl-2 member in the presence of Taxol accelerated DiM in sensitive HeLa and HaCaT cells (Figure 3B; Supplementary Table S5, S6) for both cell lines time spent in mitosis until death shortened twice in the presence of A-1155463, A-1331852, or navitoclax and shortened to a lesser extent in the presence of venetoclax or S63845.

To test whether inhibition of Bcl-xL sensitizes cells to apoptosis specifically during mitotic arrest, we used each Bcl-2 inhibitor in combination with staurosporine. After treatment with staurosporine only, MOMP was observed on average after 11 h in A549 cells, 10 h in PC-3 cells, 5 h in HaCaT cells, and 4 h in HeLa cells (Figure 3B). Timings of MOMP corresponded to the difference in sensitivity during mitotic arrest for these four cell lines, with A549 and PC-3 being more resistant to staurosporine-induced apoptosis than HaCaT and HeLa cells. However, when staurosporine was used with each inhibitor of Bcl-2 proteins, timings for MOMP remained almost unchanged for HaCaT and HeLa cells and decreased three times for A549 and PC-3, resembling those observed in HaCaT and HeLa (Figure 3B).

Since inhibition of Bcl-xL effectively sensitized cells to DiM and accelerated MOMP in resistant cell lines, we assumed that duration of mitotic arrest could depend on the activity of Bcl-xL within the cell. To test this, we treated A549 and PC-3 cells with 1 µM doses of nocodazole, Taxol, or vinorelbine in combination with 10–10000 nM doses of A-1155463, A-1331852, or navitoclax. Mitotically arrested A549 and PC-3 cells were sensitized to apoptosis under treatment with A-1155463, A-1331852, or navitoclax in a dose-dependent manner (Figure 3E; Supplementary Figure S3, S5 and Supplementary Figure S7-S9). In both cell lines, with increasing dose of A-1155463, A-1331852, or navitoclax cell fates shifted from survival after mitotic slippage to death after mitotic slippage and then to DiM. The effect of Bcl-xL inhibition on survival of mitotic cells was evident starting from 10–30 nM and 100% of DiM was observed at 300–1000 nM of Bcl-xL inhibitors. At maximal doses of Bcl-xL inhibitors (3–10 µM), average duration of mitotic arrest in slippage-prone cell lines also decreased significantly (Figure 3E; Supplementary Figure S4, S6; Supplementary Table S7-S9).

### Sequential treatment with mitotic then anti-apoptotic inhibitors stimulates cell death during a limited time

To test whether inhibition of Bcl-xL would specifically affect cell survival after prolonged mitotic arrest, we applied sequential treatments with MT drugs and A-1155463. The cells were first pre-incubated in 1 µM of nocodazole, Taxol, or vinorelbine for 24 h. Then MT drugs were removed from the media and cells were placed on the microscope stage for time-lapse observation. During the image acquisition, 4 h or 20 h after removal of MT drugs, 1 µM A-1155463 was added, whereas another set of wells remained in fresh culture medium as a control group. When removal of MT drugs was not followed by addition of A-1155463, > 90% of mitotic cells underwent mitotic slippage or division and survived for at least 48 h. After nocodazole treatment (N = 67 cells), 26 cells underwent mitotic slippage within 4.54 ± 2.87 h, and 41 cells divided within 2.40 ± 1.06 h. After Taxol treatment (N = 77 cells), 29 underwent mitotic slippage within 7.16 ± 2.62 h, and 48 cells divided abnormally within 6.05 ± 2.28 h. All cells except one survived. After vinorelbine treatment (N = 58 cells), 21 cell underwent mitotic slippage within 9.57 ± 5.03 h, and 37 cells divided normally within 8.33 ± 4.00 h. All cells except one survived.

The addition of Bcl-xL inhibitor 4 h after removal of MT drug significantly sensitized mitotic and post-mitotic cells to death. Furthermore, 50% of cells that were in mitosis at the moment of A-1155463 addition died in mitosis, whereas 50–90% of cells that underwent slippage before addition of A-1155463 died in interphase soon after addition of the drug (Figure 3F). The addition of Bcl-xL inhibitor 20 h after removal of MT drug was less efficient. The results are summarized in Supplementary Table S13. The strong effect (high rate of cell death) is achieved when A-1551643 is added 4 h after Taxol and vinorelbine treatments, and the moderate effect (approximately 50% of cell death) is achieved when A-1551643 is added 4 h after nocodazole treatment and 20 h after Taxol and vinorelbine treatments. The addition of A-155643 20 h after nocodazole treatment gives no effect at all. Similar results were obtained using A-1331852 after the same treatments (data not shown).

We conclude that sequential use of mitotic inhibitors and inhibitors of Bcl-xL anti-apoptotic protein will be efficient only if Bcl-xL inhibitor is added before cells come out of mitotic arrest or within short time after slippage.

### Inhibition of caspases prevents DiM in most cells but do not prevent MOMP

Inhibition of caspases with pan-caspase inhibitor Z-VAD(OH)-FMK has been reported to block DiM and promote mitotic slippage in some cell lines (Gascoigne and Taylor, 2008; Huang et al., 2010), but not in others (Niikura et al., 2007).

**FIGURE 4:**
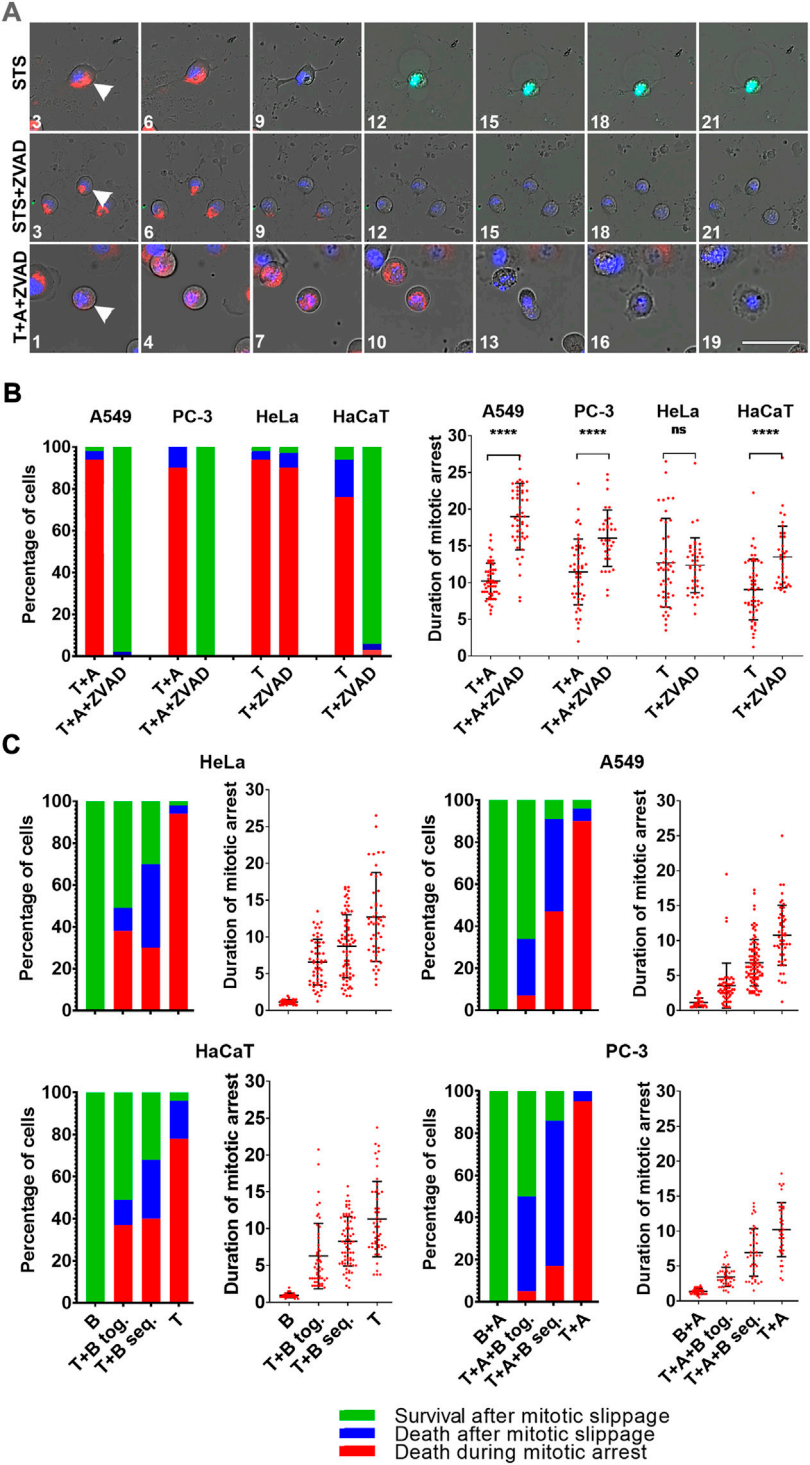
**(A)** Time-lapse sequences of A549 cells treated with staurosporine (STS), staurosporine + Z-VAD(OH)-FMK (STS + ZVAD), or Taxol + A-1155463 + Z-VAD(OH)-FMK (T + A + ZVAD). For staurosporine treatment, numbers indicate hours since drug addition. For Taxol treatment numbers indicate hours since mitotic entry. Mitochondrial membrane potential is shown in red, caspases 3/7 in green, and DNA in blue. Images were obtained using ×20/0.8 objective. Scale bar = 50 µm. **(B)** Distribution of fates and average durations of mitotic arrest (in hours) of A549 and PC-3 cells that were sensitized to DiM with 10 µM A-1155463**(A)** and DiM-sensitive HeLa and HaCaT cell lines after treatment with 1 µM Taxol (T) in the absence and presence of 100 µM pan-caspase inhibitor Z-VAD(OH)-FMK (ZVAD). **(C)** Dependence of the outcome of mitotic arrest on its duration in DiM-sensitive HeLa and HaCaT cells, and A549 and PC-3 cells that were sensitized to DiM with 10 µM A-1155463 **(A)**. For each cell lines, the longest average duration of mitotic arrest was obtained after treatment with 1 µM Taxol (T) (+A for A549 and PC-3) and the shortest with 300 nM barasertib **(B)** (+A for A549 and PC-3). Duration of mitotic arrest was manipulated using simultaneous treatment with Taxol and barasertib (T+(A+) B tog.) or sequential treatment with Taxol followed by addition of barasertib 24 h later (T+(A+)B seq.).

Addressing the question, whether inhibition of caspases can prevent MOMP during mitotic arrest and whether cells can undergo mitotic slippage after MOMP we stained DiM sensitive HeLa and HaCaT cells with TMRE and caspases 3/7 detector, and treated them with 1 µM Taxol in combination with 100 µM Z-VAD(OH)-FMK. The same experiment was performed on A549 and PC-3 cells sensitized to DiM by A-1155463 treatment (Figure 4A).

Inhibition of caspases did not prevent MOMP but completely inhibited caspases 3/7 activation in all cases, but the final response of HeLa cells was different from others. Inhibition of caspases did not prevent DiM in HeLa cells that happened similarly to the cells arrested in mitosis in the absence of caspase inhibitor (Figure 4B). Same treatment of three other cell lines prevented DiM. It resulted in prolonged mitotic arrest and >90% of mitotic cells underwent mitotic slippage after MOMP and survived for at least 24 h after mitotic slippage without recovery of mitochondrial membrane potential (Figure 4B; Supplementary Table S11).

### Shortening the duration of mitotic arrest in DiM sensitive cells increases the probability of cell survival

Since inhibition of Bcl-xL accelerated onset of MOMP during mitotic arrest in resistant A549 and PC-3 cells, we assumed that terminating mitotic arrest by inhibiting SAC activity would prevent MOMP during mitotic arrest in DiM sensitive HeLa and HaCaT cells and A549 and PC-3 cells that were sensitized to DiM by additional treatment with 10 µM of A-1155463. We used Aurora B kinase inhibitor Barazertib (AZD 1152-HQPA) as a potent drug that silences SAC (Ditchfield et al., 2003). Effective concentrations of barasertib have been previously reported for some cell lines (Helfrich et al., 2016). We used the reported concentrations as a starting point and then determined minimal efficient concentration for each of our cell lines by titrating barasertib upwards and downwards with a 3-fold step. The minimal efficient concentration of Barazertib inducing rapid mitotic slippage in 100% of cells was determined for HeLa cells (300 nM), HaCaT (1000 nM), A549 (100 nM) and PC-3 (100 nM). When barasertib was applied in the aforementioned concentrations, cells from each cell line underwent mitotic arrest followed by rapid mitotic slippage (within 60–90 min) and then survived for >24 h (Figure 4C). Flow cytometry confirmed that many cells in the presence of barasertib were able to undergo second round of DNA replication within 48 h (Supplementary Figure S10).

By manipulating the duration of mitotic arrest using simultaneous and sequential treatment with Taxol and barasertib, we showed that probability of cell survival decreased with prolonging the duration of mitotic arrest and artificially sensitized A549 and PC-3 were more prone to death after slippage rather than DiM (Figure 4C; Supplementary Table S11, S12).

### Kinetics of death in mitosis and death after slippage are different

**FIGURE 5:**
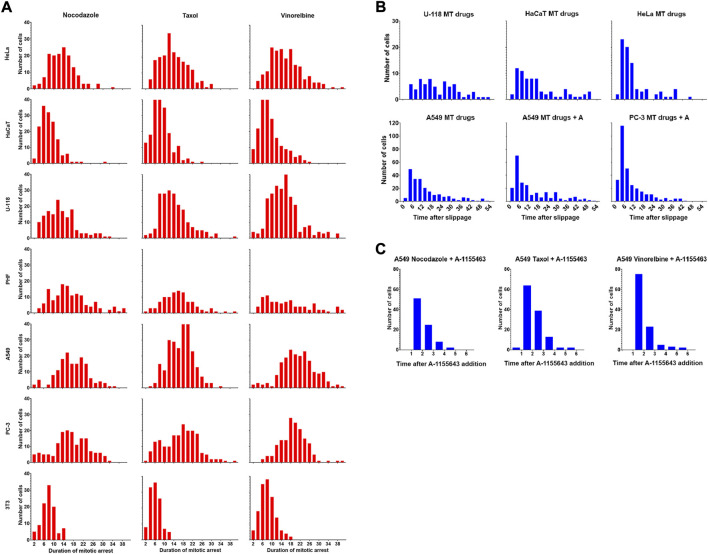
**(A)** Drug-dependent frequency distributions of the duration of mitotic arrest for seven cell lines. Time is shown in hours **(B)**. Frequency distributions of the duration of post-slippage interphase for the cells that died after mitotic slippage. Data is shown for U-118, HaCaT, HeLa, and A549 cells lines (MT drugs) and for sensitized A549 and PC-3 (MT drugs + A1155463). Data for each cell line are accumulated for three drugs. Time is shown in hours **(C)**. Frequency distributions of the duration of post-slippage interphase for A549 cells that died after mitotic slippage after sequential treatment with MT drugs and A-1155463.

Since overall survival after mitotic arrest in some cases largely depends on the death after slippage (DaS), we analyzed its kinetics in more detail. The frequency distributions for the duration of mitotic arrest are always close to normal both in DiM-sensitive and slippage-prone cell lines (Figure 5A). However, distribution of time intervals between the moment of mitotic slippage and the moment of death after slippage are asymmetric in A549 and HeLa cells demonstrating rapid decay with time (Figure 5B). Time intervals for U-118 and HaCaT cells show more broad distributions.

When A549 and PC-3 cells were partially sensitized to DiM by A-1155463, relative frequency of DaS also increased (Figure 4C), allowing to obtain more detailed information about its kinetics. The distribution of time intervals between the moment of mitotic slippage and the moment cell death was strikingly asymmetric there (Figure 5B). Frequency distribution of death after slippage when A-1155643 was added after removal of anti-mitotic drugs decayed similarly (Figure 5C).

## Discussion

### Systematic approach to inter- and intra-line variation: T1 and T2 doses

To systematically assess the degree of variation of cell fates in response to MT drugs described previously (Gascoigne and Taylor, 2008), we determined reference doses (T1 and T2) for each cell line-drug type pair by treating cell lines with a wide range of doses (1–1000 nM) of 3 MT drugs. The maximal degree of cell fates variation described previously (Gascoigne and Taylor, 2008) is observed between T1 and T2 doses. We for the first time show that above the T2 dose variation in cell responses decreases and is limited by three outcomes: death in mitosis, death after slippage and prolonged survival. We show that increasing drug concentration above T2 level does not results in decreased cell survival.

According to the response to MT drugs above T2 level cell lines could be described as DiM-sensitive and slippage-prone. DiM sensitive cell lines show large accumulation of sub-G1 population after 48 h treatment with all anti-mitotic drugs. As shown previously this population could be heterogeneous including viable cells however sub-G1 cells show limited lifespan (Potashnikova et al., 2019). Slippage-prone cell lines are characterized by accumulation in the G2/M population and sometimes show increase of tetraploid population. Besides, certain portion of these cells is accumulated in the sub-G1 population that is in accord with death in mitosis and death after slippage observed in such cells. It should be noted that maximal doses of MT drugs often result in the decrease of sub-G1 population compared to the intermediate doses.

Testing the hypothesis that frequency of DiM depends on the duration of mitotic arrest (Huang et al., 2010; Bennett et al., 2016) and based on our data that outcomes of mitotic arrest are independent from the drug used we determined the outcome of mitotic arrest for DiM sensitive cells reducing duration of the mitotic arrest by combined treatments with Taxol and barasertib. The results obtained on HeLa, HaCaT and DiM-sensitized A549 and PC-3 cells are in line with previous observations (Huang et al., 2010; Bennett et al., 2016). Besides, we observed that when duration of mitotic arrest is intermediate for a given cell line instead of DiM the frequency of DaS increases.

### MOMP is the defining step for apoptosis during mitotic arrest

Large body of evidence suggests that cell death during mitotic arrest and after mitotic slippage proceeds *via* mitochondria dependent apoptotic pathway through p53 activation (Gascoigne and Taylor, 2008; Orth et al., 2012; Jun and Kim, 2016; Pedley and Gilmore, 2016; Yasuhira et al., 2016; Ruan et al., 2019) or through extrinsic pathway (Chang et al., 2009; Pollak et al., 2021). Execution of both apoptotic pathways converges to MOMP (mitochondrial outer membrane permeabilization) and further proceeds through activation of executive caspases (Vorobjev and Barteneva, 2015; Vorobjev and Barteneva, 2016; Kalkavan and Green, 2018). Our previous studies showed that surrogate probe for MOMP could be used to follow rapid decline of mitochondrial membrane potential (TMRE staining) along with cell shrinkage (Vorobjev and Barteneva, 2015, 2016). During apoptosis execution after MOMP the activation of executive caspases 3/7 happens several hours later and can be followed using caspases 3/7 specific reagent (Vorobjev and Barteneva, 2015; Vorobjev and Barteneva, 2016; Widden and Placzek, 2021).

In our experiments, tracking the dynamics of MOMP and caspase activity provided detailed insights into the events taking place during the mitotic arrest or post-slippage interphase at a single cell level. We were able to show that MOMP during mitotic arrest is always followed by DiM, while mitotic slippage is possible only with the normal mitochondrial potential. This approach allowed us to describe in detail the effect of caspase inhibition during mitotic arrest.

Inhibition of caspases has been reported to block DiM, prolong the duration of mitotic arrest and induce mitotic slippage for some cell lines (Gascoigne and Taylor, 2008; Shi et al., 2011), but not in others (Niikura et al., 2007). To analyze this discrepancy in detail we used TMRE/caspases 3/7 reagent staining and were able to determine that inhibition of caspases did not inhibit MOMP, that is, the first visible step of apoptosis happens in the absence of caspase activity. Caspase inhibitor allowed DiM-sensitive HaCaT and artificially sensitized A549 and PC-3 cells to undergo mitotic slippage followed by prolonged survival even when MOMP occurred during mitotic arrest. Whereas HeLa did not survive in these conditions and underwent DiM after caspase inhibition at the same time as without caspase inhibitor. The contradiction in the response of cells arrested in mitosis to Z-VAD(OH)-FMK treatment becomes now apparent, since for some cells MOMP alone is sufficient to induce caspase-independent apoptosis through leakage of Smac-Diablo, IAP and Endonuclease G (Chipuk et al., 2006; Niikura et al., 2007; Zhang et al., 2017), while other cells can survive after MOMP for more than 48 h (Lartigue et al., 2009).

### Bcl-xL is the major regulator of cell death during mitotic arrest

Our analysis using highly specific Bcl-2 inhibitors shows that while all three members of Bcl-2 family play similar roles in STS-induced apoptosis, only Bcl-xL acts as the major regulator of cell death during or after mitotic arrest. Inhibition of Bcl-xL with relatively low doses (1 uM) of A-1155643 was sufficient to direct all cells from DiM resistant cell lines to apoptosis during mitotic arrest or after mitotic slippage.

It is important to notice that Mcl-1 also was shown to be a prominent regulator increasing sensitivity to mitotic arrest in primary mouse fibroblasts and several carcinoma cell lines sensitive to DiM due to its degradation during mitotic arrest (Harley et al., 2010; Wertz et al., 2011; Colin et al., 2015; Haschka et al., 2015). However, observations on increasing sensitivity to DiM after inhibition or down-regulation of Mcl-1 in cells prone to mitotic slippage were lacking. Our direct observations using specific inhibitors demonstrate that only normal human fibroblasts are sensitive to inhibition of Mcl-1, while four cancer cell lines are not. We suggest that important role is played by Mcl-1 only when the level of Bcl-xL is relatively low.

During mitotic arrest strong phosphorylation of Bcl-xL in PC-3 and HaCaT cells but almost no phosphorylation in A549 cells occurs (Supplementary Figure S4). Taking into account similarities in the responses of these two slippage-prone cell lines (A549 and PC-3) to Bcl-xL inhibitors we cannot confirm that Bcl-xL phosphorylation is important for priming to apoptosis during mitotic arrest. Inhibition of Bcl-xL did not induce apoptosis during normal mitosis or abnormal division followed by rapid mitotic slippage (see also Bennett et al., 2016), suggesting that apoptosis is only initiated in the mitotic cells arrested for prolonged time (>3 h). Thus, manifestation of one of three possible fates (cell survival, DiM, or DaS) depends on the initial activity of Bcl-xL (see the model below).

Prolonged follow-up of cells after mitotic slippage clearly shows that post-mitotic response leading to senescence followed by subsequent cell death for cancer cell lines is a slow process and cell death is delayed for >24 h (Ditchfield et al., 2003; von Zglinicki et al., 2021). Kinetics of death after slippage observed in our experiments shortly (Figure 5) deserves another explanation. Probability of death after slippage shows nearly exponential decay with time indicating that the trigger signal is at maximal level immediately after slippage. We assume that DaS is partially triggered during mitotic arrest as suggested by Gascoigne and Taylor (2008) making arrested cells primed to apoptosis (Terrano et al., 2010; Bah et al., 2014; Tao et al., 2014; Pedley and Gilmore, 2016) and MOMP as a final step committing cell to apoptosis proceeds after slippage in a stochastic way. The final step might be triggered by DNA damage response (DDR) activation in the interphase nuclei due to DNA damage accumulated during prolonged mitotic arrest (Orth et al., 2012). During mitotic arrest DNA reparation process that is partially inhibited (Clay and Fox, 2021) and it is activated at the beginning of interphase. The signal triggering apoptosis after slippage that comes at the onset of DDR is quickly downregulated during interphase because of DNA reparation and for cells have been survived after slippage for several hours the probability to die decreases with time.

### Cell fate depends on Bcl-xL activity and duration of mitotic arrest

The model of competing networks proposed by Gascoigne and Taylor (2008) explains cell response to mitotic arrest in a binary fashion and does not explain DaS, which is often observed after prolonged mitotic arrest in several cell lines (A549, U118, and HaCaT).

**FIGURE 6:**
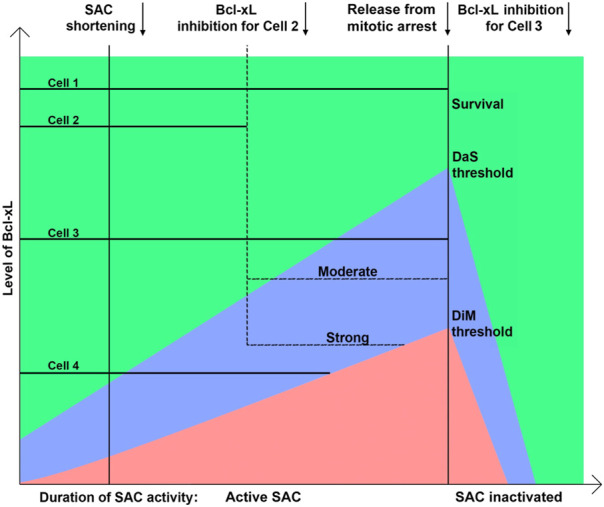
Model explaining the regulation of the outcome of mitotic arrest by the Bcl-xL level and duration of mitotic arrest. After release from mitotic arrest cells divide or undergo mitotic slippage and rapidly restore anti-apoptotic proteins activity. Death after slippage area is shown in blue; death in mitosis is shown in red. Cells’ fates can be shifted from survival to death in mitosis or death after slippage by inhibiting Bcl-xL to a certain level.

Thus, we propose a modified model that describes all outcomes of mitotic arrest based on the pro-apoptotic activity increasing with time, intra-cellular level of Bcl-xL and duration of SAC activity (Figure 6). When the level/activity of Bcl-xL is low, or decreased by inhibitor, elevating pro-apoptotic signal induces DiM before sufficient amount of CyclinB1 is degraded to allow mitotic exit.

In our experiments, inhibition of Bcl-xL in the cells that were arrested in mitosis for more than 4 h induced rapid DiM within 2 h after inhibition of Bcl-xL, whereas inhibition of Bcl-xL from the beginning of mitotic arrest induced DiM only as early as 6–7 h. It suggests the presence of pro-apoptotic activity elevating with time during mitotic arrest that increases the probability of apoptosis resulting in DiM (red area) or DaS (blue area) and decreases the probability of survival (green area) (Figure 6). On the other hand, Bcl-xL appears to be the main anti-apoptotic factor during mitotic arrest that prevents MOMP. Higher amount of Bcl-xl is associated with increased probability of cell survival following mitotic arrest. Rate of CyclinB1 degradation defines the duration of SAC activity and acts as an independent timer for mitotic exit (Van Delft et al., 2006; Huang et al., 2010). Termination of SAC soon after mitotic entry inhibits elevation of pro-apoptotic activity and results in mitotic exit with following cell survival.

When Bcl-xL is in excess, timely degradation of CyclinB1 results in mitotic exit and prolonged survival, whereas DaS is observed when the level of Bcl-xL is high enough to allow mitotic exit but not enough to maintain the early period of post-slippage interphase. The choice between survival and DaS depends on the level of Bcl-xL at the moment of slippage. When Bcl-xL level is rather low probability of MOMP and subsequent cell death is highly probable due to activation of DDR. When this level is significantly above the threshold, probability of MOMP is low and rapid recovery after slippage makes MOMP highly unlikely within few hours.

Taking into account results of treatments of slippage-prone cells with Bcl-xL inhibitor we conclude that cell fate after slippage depends on the balance of pro-apoptotic signaling and anti-apoptotic defense relies mainly on the Bcl-xL activity.

The duration of mitotic arrest in the resistant cells is reduced only by treatment with Bcl-xL inhibitors (except HeLa cells). Inhibitors of Bcl-2 and Mcl-1 can increase the frequency of DiM of normal fibroblasts (but not of cancer cells) but duration of mitotic arrest does not change in these cases. Inhibitors of Bcl-xL have double effect–increase in the frequency of DiM and reducing duration of mitotic arrest. In DiM sensitive HaCaT cells inhibitors of Bcl-xL also reduce duration of mitotic arrest, while for HeLa cells with almost no expression of Bcl-xL inhibitors of this protein consequently show no effect.

### Can Bcl-xL inhibitor in combination with antimitotic drugs enhance efficacy of anti-tumor therapy?

The taxanes and vinca alkaloids are widely used chemotherapy agents against different tumors. However, their effect might be short-term followed by relapse of the resistant clone. Besides, some patients demonstrate resistance to anti-microtubule drugs initially. Studies on the cultured cells demonstrate that resistance comes from frequent mitotic slippage (Shi et al., 2011; Gascoigne and Taylor, 2008; current study). To overcome taxane or vinorelbine resistance additional treatment might be beneficial. Specific effect might be achieved whenever secondary drug will selectively kill cells arrested in mitosis or soon after mitotic slippage (post-mitotic cells).

Mitotic slippage followed by cell survival and proliferation is thought to be a major reason for resistance and/or relapse.

Here, we show that Bcl-xL is a potent pro-survival factor during mitotic arrest, and its inhibitors A-1155643 and A-13331852 enhanced apoptosis by both elevating frequency of death in mitosis and accelerating it as well as inducing cell death soon after mitotic slippage. These observations corroborate with previous reports on navitoclax (Chen et al., 2011; Shi et al., 2011; Tan et al., 2011; Bah et al., 2014). However, navitoclax is not specific–it also inhibits Bcl-2 (Shi et al., 2011) and induces significant side-effects in humans killing proliferating hematopoietic cells (Gandhi et al., 2011; Gupta et al., 2021; Nor Hisam et al., 2021). Our data show that nanomolar concentrations of A-1155643 and A-13331852 have the same efficacy as navitoclax on cultured cancer cells inducing DiM in highly resistant A549 and PC-3 cells. Thus, using specific Bcl-xL inhibitor might be sufficient for nearly 100% cell death after mitotic arrest.

Another question is about efficacy of Bcl-xL inhibitors in the presence of low doses of Taxol. Low concentrations of Taxol (below 10 nM) did not induce prolonged mitotic arrest in our studies (except HeLa cells–Supplementary Table S1). Since Bcl-xL inhibitors become effective only when duration of mitotic arrest is more than 4 h, we do not expect that these inhibitors will have significant effect when cells will not be arrested in mitosis. Our flow cytometry data show that no changes in the cell cycle distribution is observed when cells are treated with 1 uM of A-1155463 or A-1331852 together with Taxol in concentrations 1 and 3 nM (data not shown).

Together with the previous observations (Huang et al., 2010; Bennett et al., 2016) and taking into account that patients unresponsive to therapy using taxanes often have high expression of Bcl-xL (Wong et al., 2012; Maloney et al., 2020; Wang et al., 2020), our data prompt for exploring specific Bcl-xL inhibitors in combination with taxanes and vinca alkaloids in order to enhance antimitotic chemotherapy. Since such combinations might be extremely toxic (Bennett et al., 2016), determination of the minimal effective dose of each chemotherapy agent and development of a protocol for the simultaneous or sequential administration of these drugs are essential and require further research.

## Data Availability

The original contributions presented in the study are included in the article/Supplementary Material; further inquiries can be directed to the corresponding author.
