# Supplementary figures and images for: Bcl-xL activity influences outcome of the mitotic arrest
Source: Front Pharmacol. 2022 Sep 15;13:933112. doi: 10.3389/fphar.2022.933112 (PMC9520339; doi:10.3389/fphar.2022.933112)

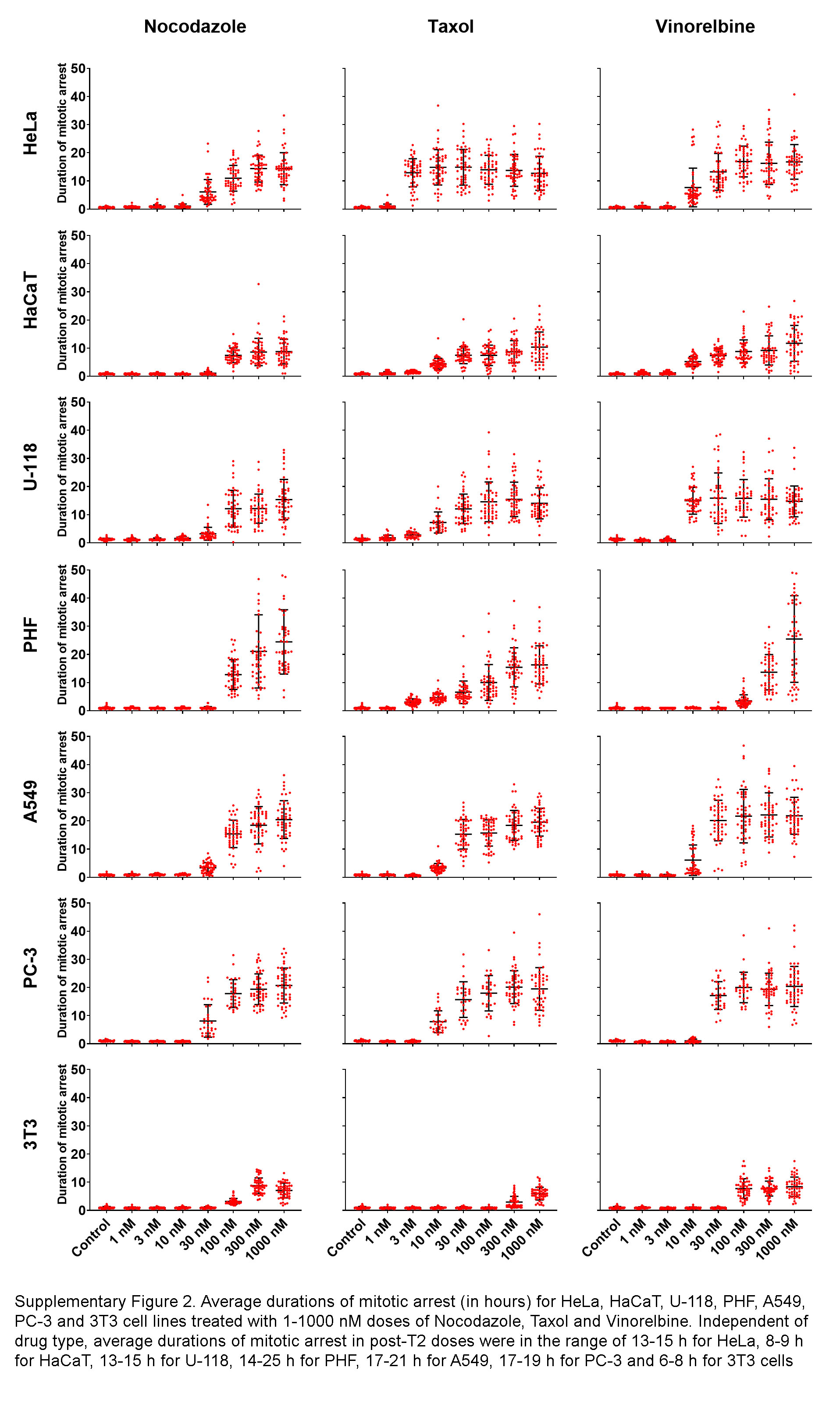

Supplement: Supplementary file 1 [file Image2.jpg]

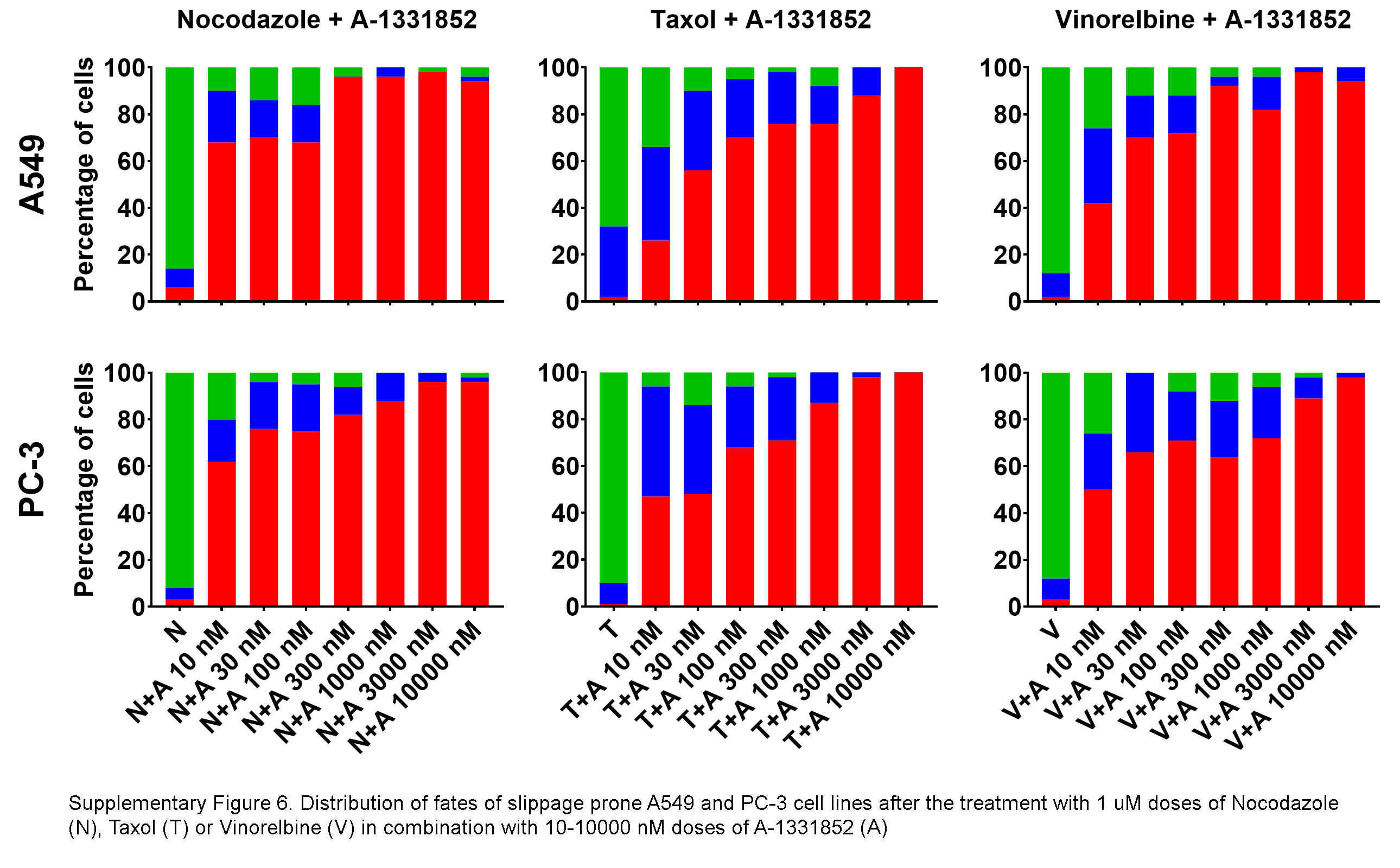

Supplement: Supplementary file 2 [file Image6.tif]

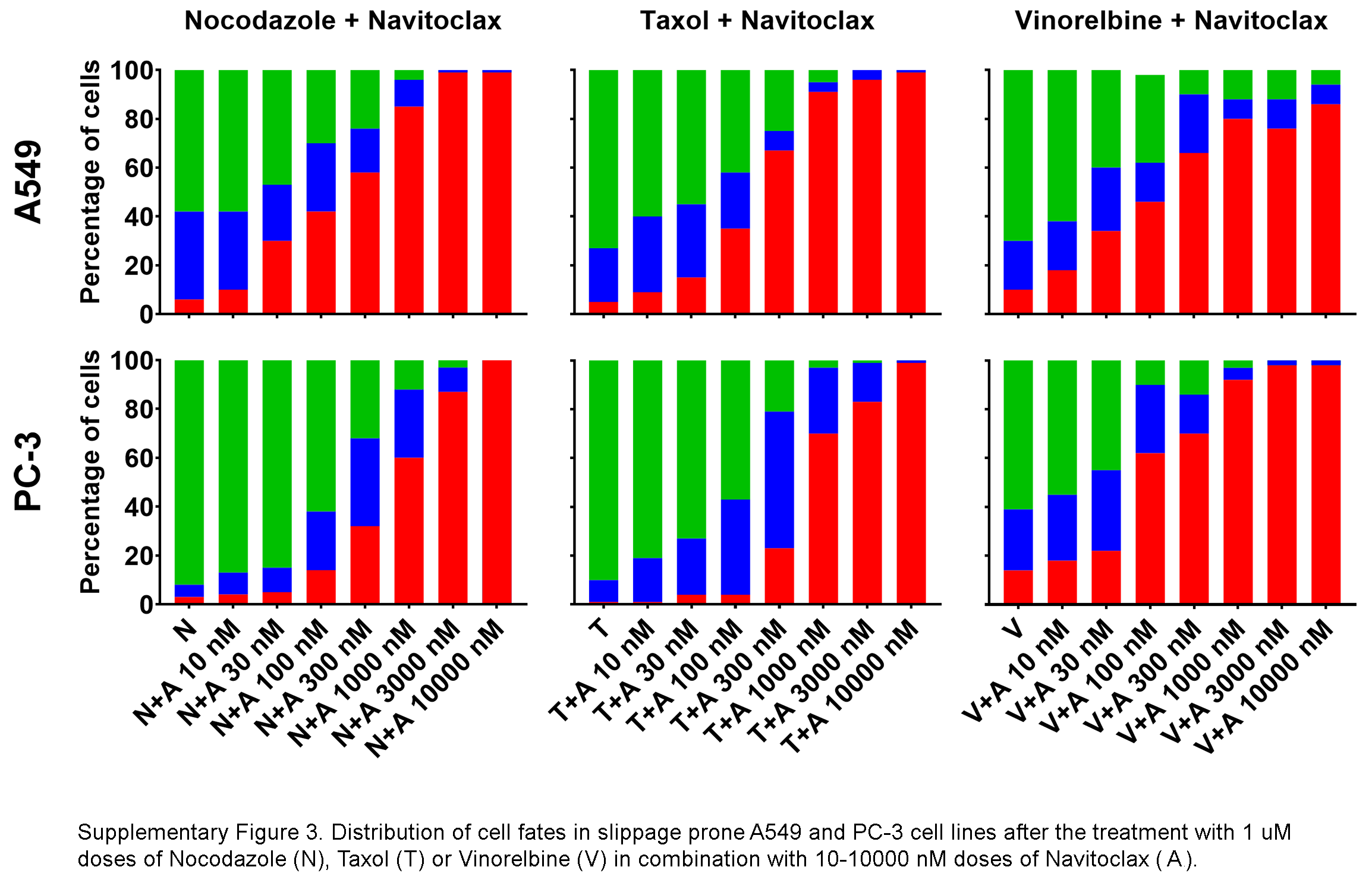

Supplement: Supplementary file 3 [file Image3.tif]

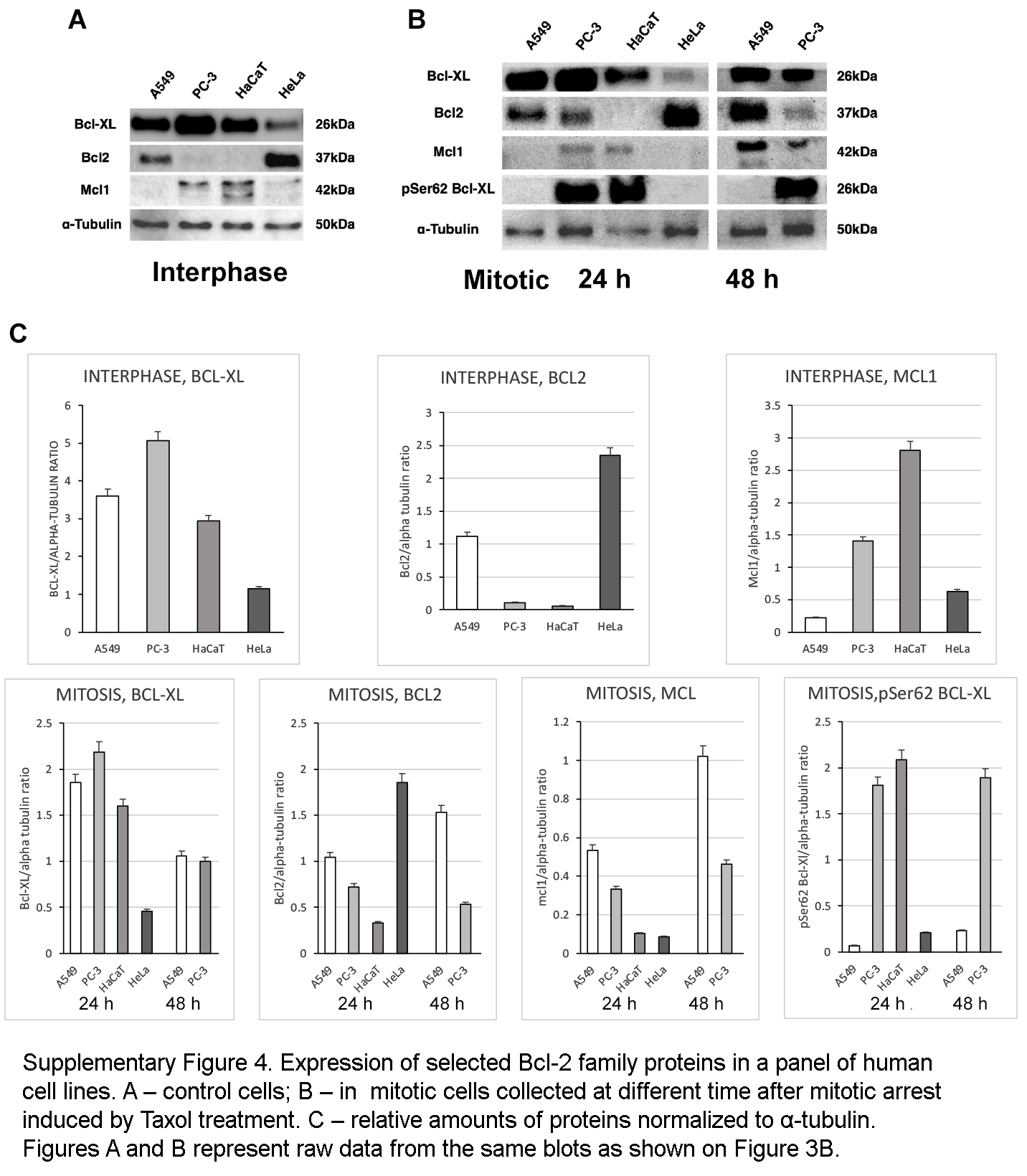

Supplement: Supplementary file 4 [file Image4.tif]

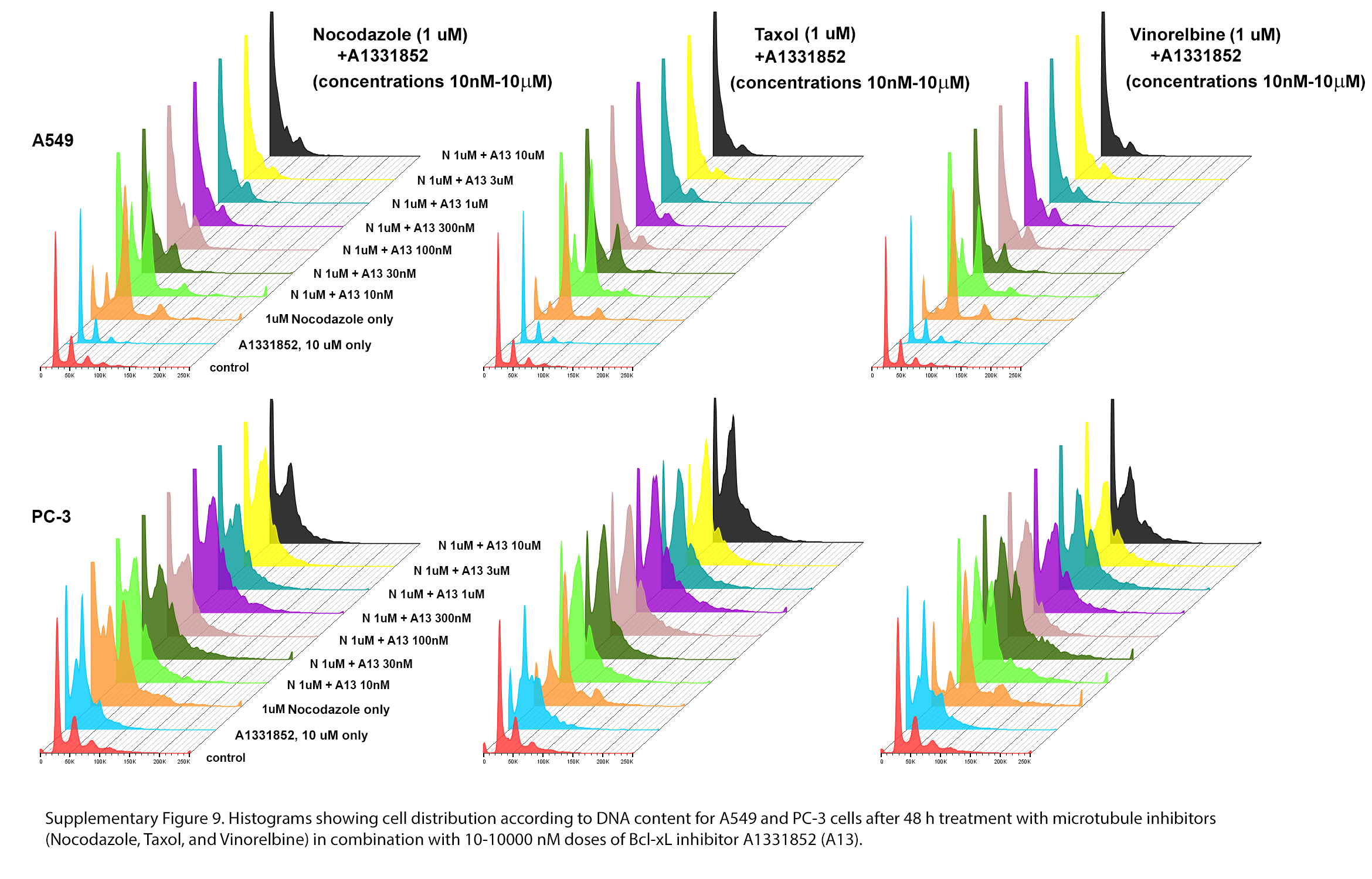

Supplement: Supplementary file 5 [file Image9.tif]

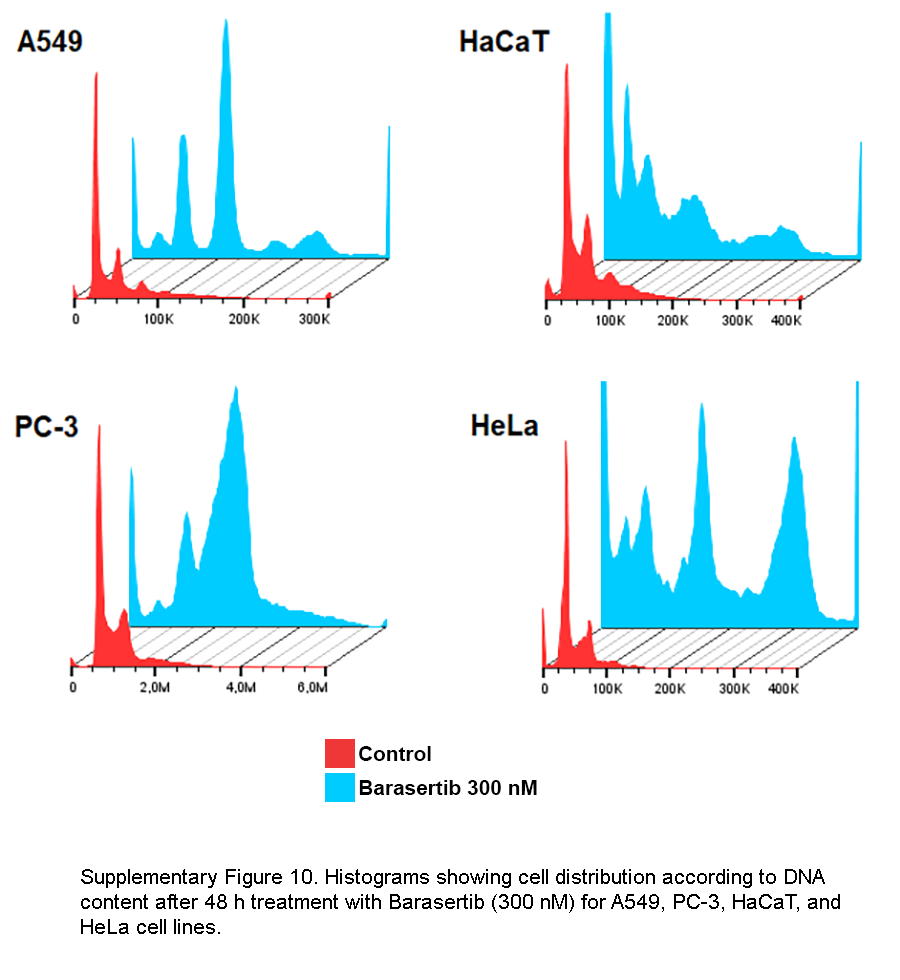

Supplement: Supplementary file 6 [file Image10.tif]

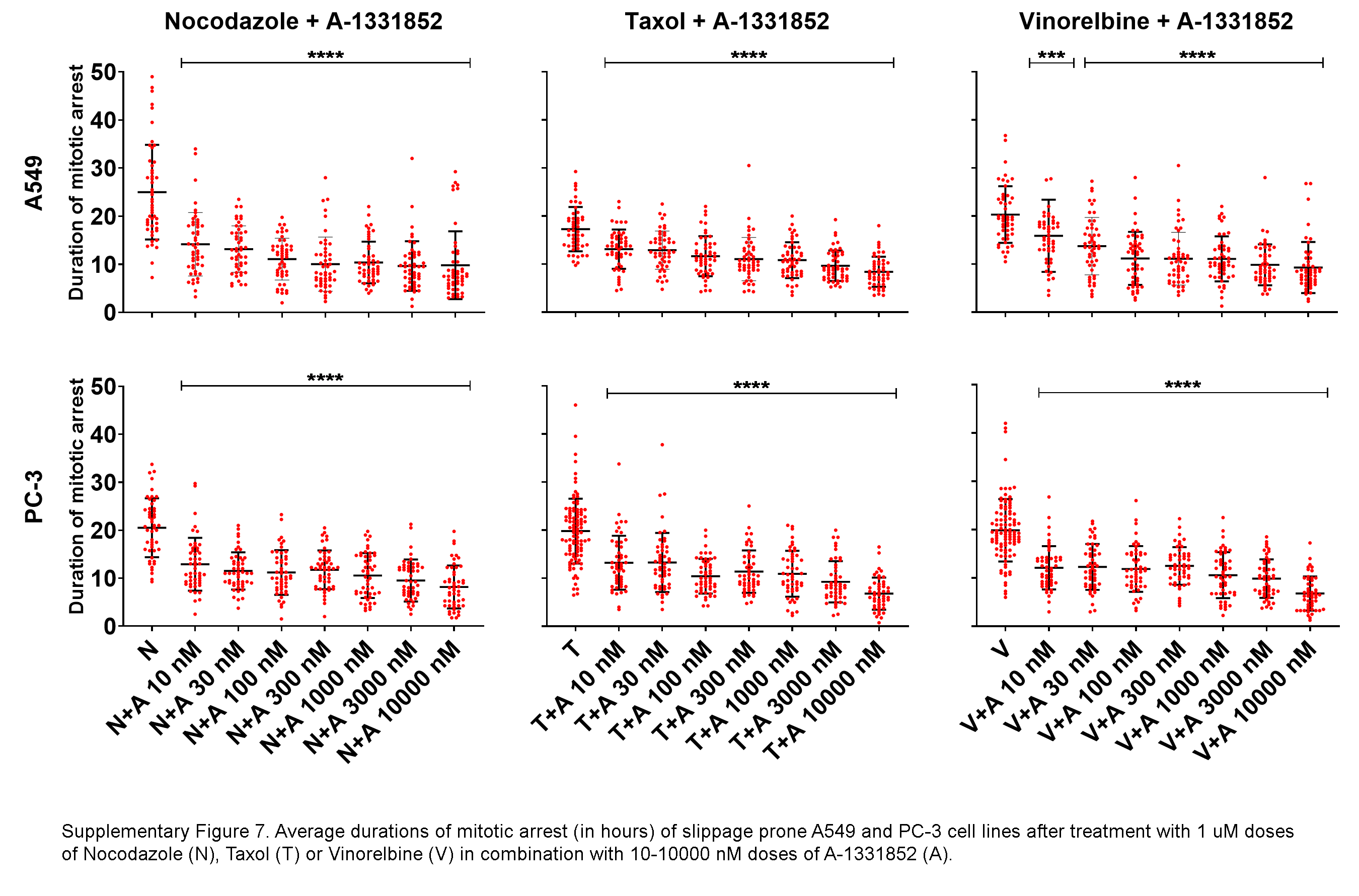

Supplement: Supplementary file 7 [file Image7.tif]

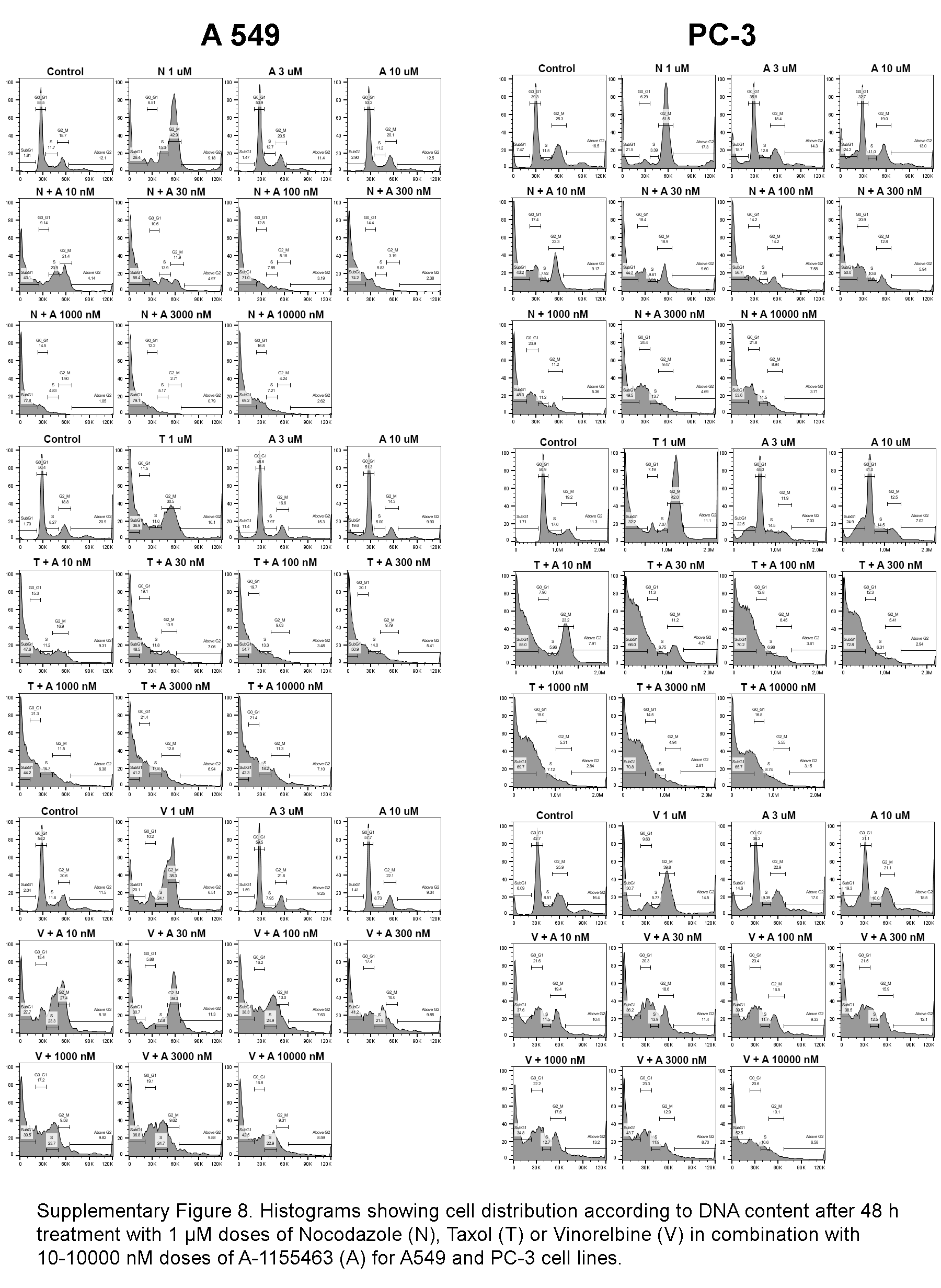

Supplement: Supplementary file 9 [file Image8.tif]

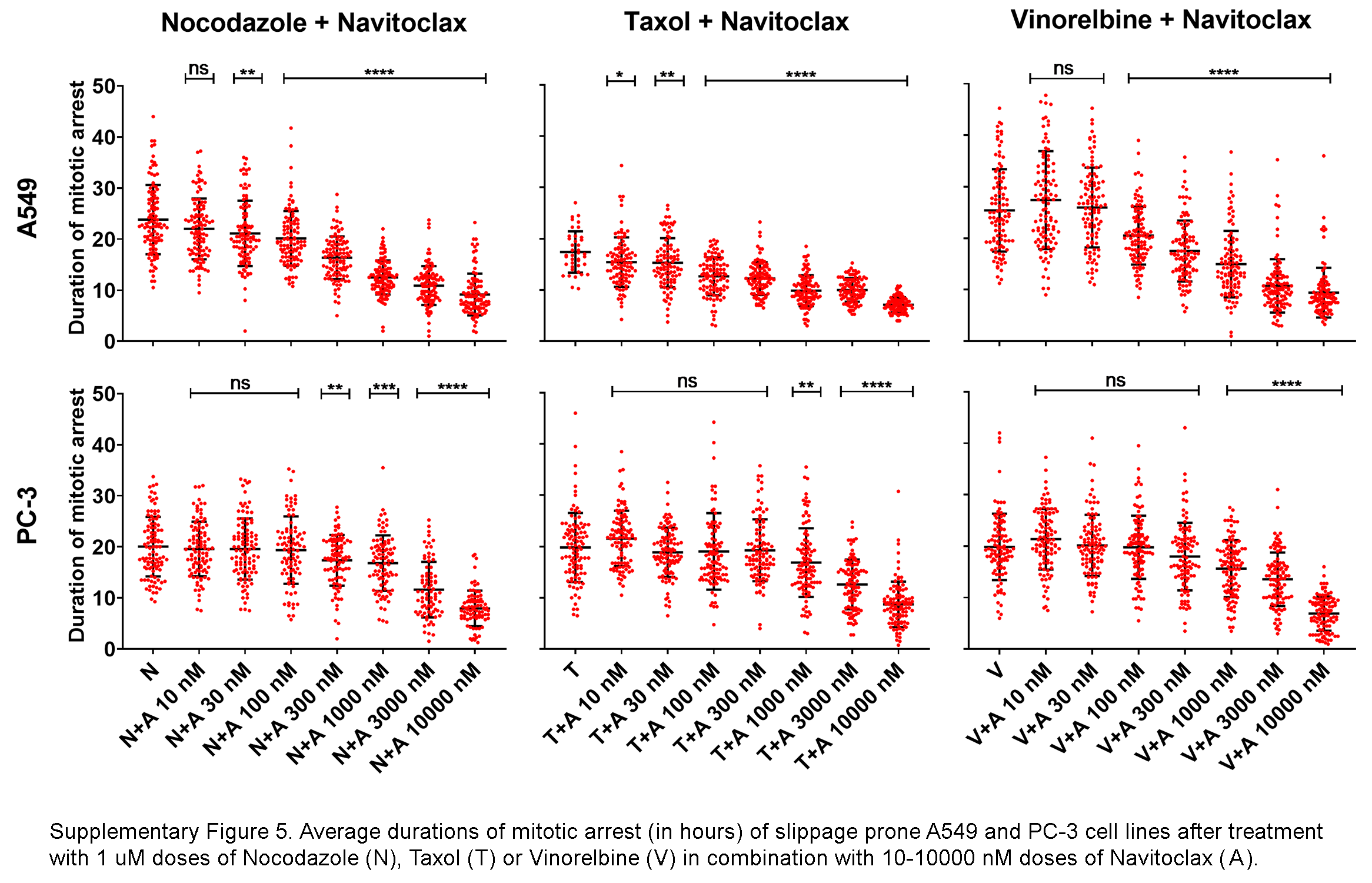

Supplement: Supplementary file 10 [file Image5.tif]

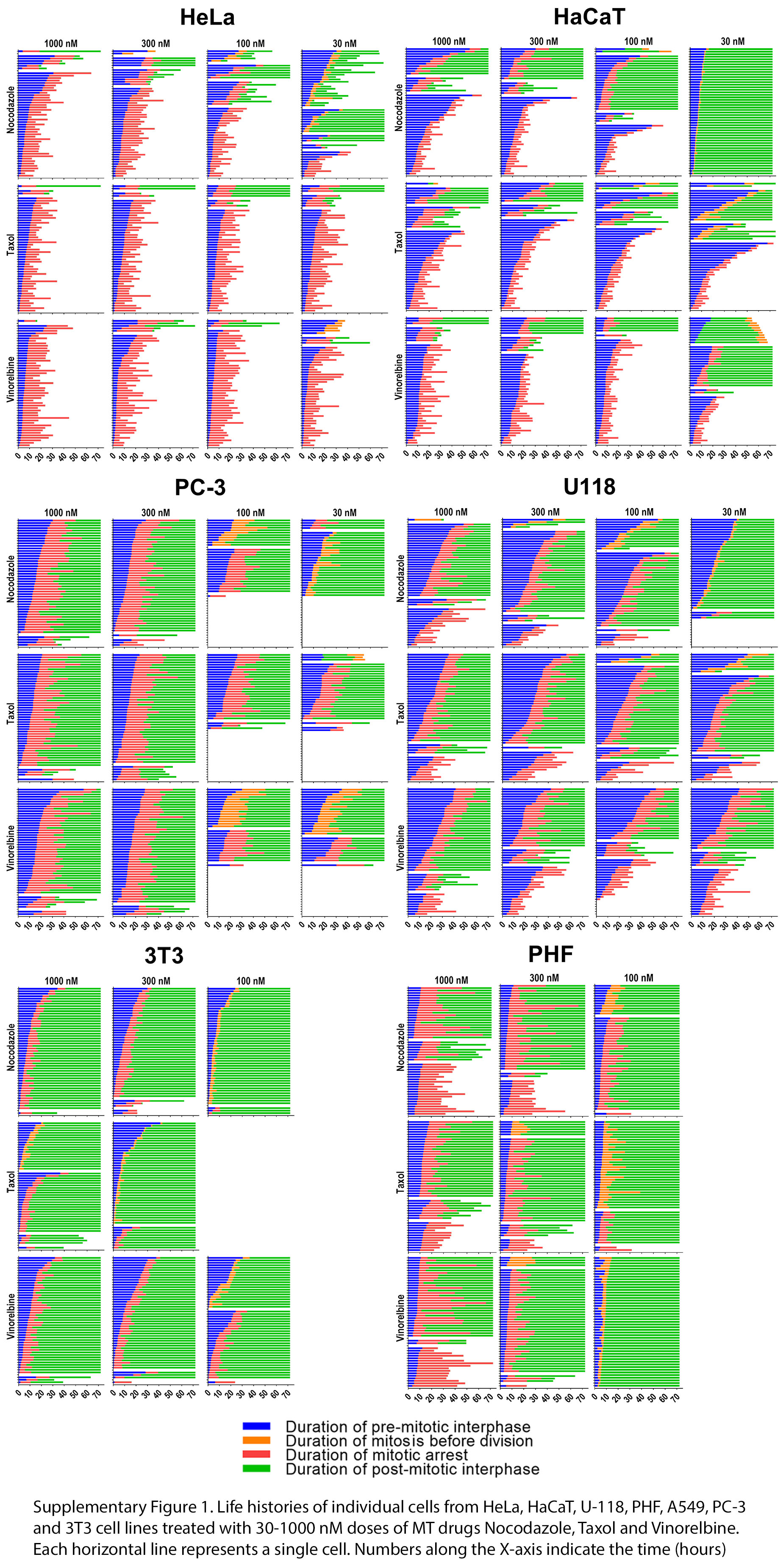

Supplement: Supplementary file 11 [file Image1.jpg]
