# Supplementary material for: Bcl-xL activity influences outcome of the mitotic arrest
Source: Front Pharmacol. 2022 Sep 15;13:933112. doi: 10.3389/fphar.2022.933112 (PMC9520339; doi:10.3389/fphar.2022.933112)
Supplement: Supplementary file 8 [file DataSheet1.pdf]

Supplementary Table 1. Average durations of mitotic stage (in hours) for HeLa, HaCaT, U-118, PHF, A549, PC-3 and 3T3 cell lines after treatment with 1-1000 nM doses of 3 MT drugs. For each cell line, T1 dose is colored in purple; durations of mitotic arrest in T2 and higher doses are colored in red (brick) for Nocodazole, green for Taxol and blue for Vinorelbine.

	Control	1 nM	3 nM	10 nM	30 nM	100 nM	300 nM	1000 nM
**HeLa**								
Nocodazole	0.64±0.22	0.76±0,30	0.98±0.53	1.08±0.74	6.09±4.41	10.97±4.58	14.38±4.77	14.34±5.69
Taxol	0.64±0.22	1.05±0.73	12.89±5.00	14.84±6.30	14.87±6.35	13.96±5.13	13.74±5.66	12.71±6.05
Vinorelbine	0.64±0.22	0.84±0.41	0.76±0.39	7.66±6.87	13.23±6.55	16.90±5.46	16.27±7.52	16.77±6.16
**HaCaT**								
Nocodazole	0.89±0.23	0.84±0.20	0.89±0.26	0.83±0.20	1,10±0.53	7.44±2.59	8.63±4.84	8.78±4.39
Taxol	0.89±0.23	1.15±0.50	1.50±0.43	4.46±1.94	7.49±3.03	7.44±3.62	8.72±3.95	10.37±5.33
Vinorelbine	0.89±0.23	1.18±0.50	1.21±0.47	5.23±1.81	7.36±2.49	8.82±4.10	9.19±5.16	11.72±6.36
**U-118**								
Nocodazole	1.22±0.40	1.18±0.46	1,30±0.44	1.58±0.65	3.20±2.30	12.12±6.50	12.15±5.14	15.36±7.17
Taxol	1.22±0.40	1.78±0.95	2.77±1.07	7.23±3.73	12.06±5.22	14.57±7.08	15.44±6.10	14.03±5.51
Vinorelbine	1.22±0.40	0.90±0.31	1.07±0.48	14.96±4.79	15.85±8.97	15.81±6.66	15.48±7.28	14.74±5.47
**PHF**								
Nocodazole	1.01±0.41	0.98±0.24	0.92±0.19	0.97±0.23	1.02±0.42	12.82±5.26	21.06±13.01	24.43±11.43
Taxol	1.01±0.39	0.93±0.22	3.08±1.17	4.61±1.6	6.55±4.01	10.03±6.36	15.41±6.95	16.28±6.77
Vinorelbine	1.01±0.39	0.87±0.19	0.89±0.15	0.97±0.18	0.97±0.36	3.48±2.17	13.65±6.27	25.45±15.37
**A549**								
Nocodazole	0.83±0.25	0,90±0.31	0.95±0.28	0.99±0.26	3.35±1.89	15.40±4.85	18.48±6.61	20.46±6.72
Taxol	0.83±0.25	0.88±0.30	0.73±0.26	3.28±1.60	15.27±5.25	15.72±4.66	18.41±5.31	19.56±4.94
Vinorelbine	0.83±0.25	0.84±0.36	0.73±0.31	6.07±5.42	20.15±7.19	21.7±9.48	22.15±7.83	21.83±6.56
**PC-3**								
Nocodazole	1.05±0.27	0.86±0.23	0.88±0.21	0.87±0.23	8.10±5.79	17.83±4.95	19.39±5.45	20.69±6.2
Taxol	1.05±0.27	0.88±0.23	0.93±0.29	7.85±3.87	15.69±6.31	17.97±6.27	20.11±5.84	19.49±7.57
Vinorelbine	1.05±0.27	0.73±0.28	0.76±0.24	1.04±0.62	17.12±4.93	20,00±5.40	19.33±5.73	20.37±7.12
**3T3**								
Nocodazole	0.98±0.37	0.88±0.30	0,90±0.25	0.97±0.28	0.96±0.28	3.20±1.05	8.77±2.74	7.06±2.62
Taxol	0.98±0.37	0.88±0.30	0.90±0.25	0.97±0.28	0.96±0.28	0.88±0.30	2.94±2.03	5.95±2.25
Vinorelbine	0.98±0.37	0.97±0.28	0.78±0.25	0.88±0.30	0.90±0.25	7.75±3.47	7.75±2.65	8.39±3.41

Supplementary Table 2. Average duration of mitotic arrest (in hours) in post-T2 doses of Nocodazole, Taxol and Vinorelbine for HeLa, HaCaT, U-118, PHF, A549, PC-3 and 3T3 cell lines. N= >100 cells for each treatment group.

	Nocodazole	Taxol	Vinorelbine
**HeLa**	13.23±5.25	13.82±5.82	15.79±6.59
**HaCaT**	8.28±4.08	8.48±4.19	9.27±4.97
**U-118**	13.21±6.47	14.02±6.1	15.26±6.71
**PHF**	22.75±12.3	15.85±6.845	19.55±13.1
**A549**	18.11±6.43	16.94±5.22	21.45±7.82
**PC-3**	17.39±7.25	16.99±7.49	19.37±6.08
**3T3**	7.91±2.8	5.95±2.35	7.96±3.19

Supplementary Table 3. Comparison of durations of mitotic arrest within the same cell line after treatment with post-T2 doses of Nocodazole (N), Taxol (T) or Vinorelbine (V). Results obtained by ANOVA analysis.

	Significant?	p-value
**N HeLa vs. T HeLa**	No	0.6302
**N HeLa vs. V HeLa**	Yes	0.0002
**T HeLa vs. V HeLa**	Yes	0.003
**N HaCaT vs. T HaCaT**	No	0.9159
**N HaCaT vs. V HaCaT**	No	0.1011
**T HaCaT vs. V HaCaT**	No	0.1784
**N U-118 vs. T U-118**	No	0.474
**N U-118 vs. V U-118**	Yes	0.0061
**T U-118 vs. V U-118**	No	0.107
**N PHF vs. T PHF**	Yes	<0.0001
**N PHF vs. V PHF**	No	0.1051
**T PHF vs. V PHF**	Yes	0.0489
**N A549 vs. T A549**	No	0.1863
**N A549 vs. V A549**	Yes	<0.0001
**T A549 vs. V A549**	Yes	<0.0001
**N PC-3 vs. T PC-3**	No	0.8565
**N PC-3 vs. V PC-3**	Yes	0.0303
**T PC-3 vs. V PC-3**	Yes	0.0044
**N 3T3 vs. T 3T3**	Yes	<0.0001
**N 3T3 vs. V 3T3**	No	0.9908
**T 3T3 vs. V 3T3**	Yes	<0.0001

Supplementary Table 4. Results from ANOVA analysis. Comparison of durations of mitotic arrest between different cell lines after treatment with post-T2 doses of Nocodazole, Taxol or Vinorelbine.

	Nocodazole		Taxol		Vinorelbine	
	Significant?	p-value	Significant?	p-value	Significant?	p-value
**HeLa vs. HaCaT**	Yes	<0.0001	Yes	<0.0001	Yes	<0.0001
**HeLa vs. U-118**	No	>0.9999	No	0.9998	No	0.9853
**HeLa vs. A549**	Yes	<0.0001	Yes	<0.0001	Yes	<0.0001
**HeLa vs. PC-3**	Yes	<0.0001	Yes	<0.0001	Yes	<0.0001
**HeLa vs. 3T3**	Yes	<0.0001	Yes	<0.0001	Yes	<0.0001
**HeLa vs. PHF**	Yes	<0.0001	No	0.0535	Yes	0.0002
**HaCaT vs. U-118**	Yes	<0.0001	Yes	<0.0001	Yes	<0.0001
**HaCaT vs. A549**	Yes	<0.0001	Yes	<0.0001	Yes	<0.0001
**HaCaT vs. PC-3**	Yes	<0.0001	Yes	<0.0001	Yes	<0.0001
**HaCaT vs. 3T3**	No	0.9995	Yes	0.003	No	0.5929
**HaCaT vs. PHF**	Yes	<0.0001	Yes	<0.0001	Yes	<0.0001
**U-118 vs. A549**	Yes	<0.0001	Yes	<0.0001	Yes	<0.0001
**U-118 vs. PC-3**	Yes	<0.0001	Yes	<0.0001	Yes	<0.0001
**U-118 vs. 3T3**	Yes	<0.0001	Yes	<0.0001	Yes	<0.0001
**U-118 vs. PHF**	Yes	<0.0001	No	0.1167	Yes	<0.0001
**A549 vs. PC-3**	No	0.9646	No	>0.9999	No	0.0742
**A549 vs. 3T3**	Yes	<0.0001	Yes	<0.0001	Yes	<0.0001
**A549 vs. PHF**	Yes	<0.0001	No	0.6723	No	0.2838
**PC-3 vs. 3T3**	Yes	<0.0001	Yes	<0.0001	Yes	<0.0001
**PC-3 vs. PHF**	Yes	<0.0001	No	0.6666	No	>0.9999
**3T3 vs. PHF**	Yes	<0.0001	Yes	<0.0001	Yes	<0.0001

Supplementary Table 5. Average durations of mitotic arrest (in hours) for A549,PC-3, HeLa and HaCaT cell lines after treatment with 1 uM Taxol (T) in combination with 10 uM Navitoclax (NVTX), Venetoclax (VNTX), S63845 (S) or A-1155463 (A). N > 30 cells for each treatment group.

	T	T+NVTX	T+VNTX	T+S	T+A-1155	T+A-1331
**A549**	16.4±4.31	7.02±1.56	16.8±6.46	15.6±5.49	10.2±2.41	8.4±3.12
**PC-3**	19.5±7.65	8.71±4.44	18±6.31	19.8±5.74	11.5±4.48	6.79±3.33
**PHF**	16.3±6.78	11.2±4.99	17.6±7.57	15.5±7.08	12.8±3.65	11.2±3.53
**HeLa**	12.7±6.04	7.29±2.72	9.1±3.38	8.18±3.56	8±3.69	6.75±3.25
**HaCaT**	9.87±5.13	5.02±2.62	7.45±2.87	8.15±2.35	3.79±2.75	4.33±1.6

Supplementary Table 6. Statistical significance of the between duration of mitotic arrest between Taxol only and Taxo +Bcl-2 inhibitor treated cells. Results from ANOVA analysis.

	T vs.T+NVTX		T vs. T+VNTX		T vs. T+S63		T vs. T+A-1155		T vs. T+A-1331	
	Significant?	p-value	Significant?	p-value	Significant?	p-value	Significant?	p-value	Significant?	p-value
**A549**	Yes	<0.0001	No	0.9875	No	0.8678	Yes	<0.0001	Yes	<0.0001
**PC-3**	Yes	<0.0001	No	0.517	No	0.9986	Yes	<0.0001	Yes	<0.0001
**PHF**	Yes	<0.0001	No	0.7019	No	0.9478	Yes	0.0115	Yes	<0.0001
**HeLa**	Yes	<0.0001	Yes	<0.0001	Yes	<0.0001	Yes	<0.0001	Yes	<0.0001
**HaCaT**	Yes	<0.0001	Yes	0.0003	Yes	0.0198	Yes	<0.0001	Yes	<0.0001

Supplementary Table 7. Average durations of mitotic arrest (in hours) of slippage prone A549 and PC-3 cell lines after the treatment with 1 uM doses of Nocodazole, Taxol or Vinorelbine in combination with 10-10000 nM doses of A-1155463.

**A-1155463**	0	10 nM	30 nM	100 nM	300 nM	1000 nM	3000 nM	10000 nM
**A549**								
Nocodazole	20.43±7.35	19.76±6.24	15.43±5.75	13.04±5.3	11.05±3.3	10.56±4.11	11.1±5.45	9.88±3.88
Taxol	19.77±4.75	14.82±4.91	13.36±4.69	13.08±3.56	10.94±3.32	11.25±3.95	11.39±2.71	9.26±3.53
Vinorelbine	22.68±6.62	18.79±5.75	16.91±5.23	14.64±5.22	13.01±3.73	10.5±4.38	10.71±3.53	9.39±3.33
**PC-3**								
Nocodazole	20.52±6.14	19.34±6.46	17.33±5.02	16.68±3.81	15.54±4.79	13.83±5.51	14.8±4.82	12.8±5.26
Taxol	19.51±7.65	16.51±5.14	15.84±5.11	14.57±4.19	14.45±5.27	16.7±5.35	13.44±4.78	11.47±4.48
Vinorelbine	20.37±7.12	17.48±5.91	17.14±6.3	14.83±4.12	14.92±5.91	14.9±5.93	12.14±4.13	10.23±5.21

Supplementary Table 8. Average durations of mitotic arrest (in hours) of slippage prone A549 and PC-3 cell lines after the treatment with 1 uM doses of Nocodazole, Taxol or Vinorelbine in combination with 10-10000 nM doses of Navitoclax.

**Navitoclax**	0	10 nM	30 nM	100 nm	300 nM	1000 nM	3000 nM	10000 nM
**A549**								
Nocodazole	23.82±6.79	22.01±5.95	21.12±6.4	20.15±5.36	16.35±4.18	12.48±3.35	10.9±3.8	9.15±4.09
Taxol	17.4±4.02	15.43±4.81	15.3±4.79	12.67±3.7	12.21±3.2	9.86±3.02	9.98±2.29	7.11±1.46
Vinorelbine	25.41±8.01	27.4±9.51	25.96±7.75	20.51±5.63	17.51±5.92	14.98±6.46	10.77±5.18	9.43±4.84
**PC-3**								
Nocodazole	20.04±5.84	19.56±5.35	19.56±5.99	19.35±6.6	17.36±4.97	16.8±5.43	11.61±5.43	7.93±3.5
Taxol	19.8±6.74	21.56±5.42	18.86±4.81	19.03±7.48	19.25±6.03	16.86±6.7	12.59±4.87	8.71±4.44
Vinorelbine	19.86±6.46	21.36±5.9	20.15±5.93	19.79±6.13	17.98±6.56	15.64±5.53	13.58±5.2	6.9±3.32

Supplementary Table 9. Average durations of mitotic arrest (in hours) of slippage prone A549 and PC-3 cell lines after the treatment with 1 uM doses of Nocodazole, Taxol or Vinorelbine in combination with 10-10000 nM doses of A-1331852.

**A-1331852**	0	10 nM	30 nM	100 nm	300 nM	1000 nM	3000 nM	10000 nM
**A549**								
Nocodazole	24.99±9.83	14.16±6.602	13.16±4.81	11.08±4.34	10.03±5.62	10.38±4.33	9.63±5.17	9.81±7.05
Taxol	17.26±4.61	13.11±4.1	12.91±3.99	11.65±4.17	11.03±4.51	10.81±3.73	9.64±3.17	8.40±3.13
Vinorelbine	20.30±5.90	15.90±7.52	13.73±5.97	11.18±5.52	11.12±5.48	11.08±4.68	9.843±4.26	9.29±5.31
**PC-3**								
Nocodazole	20.52±6.14	12.90±5.51	11.51±3.87	11.19±4.66	11.75±4.03	10.55±4.69	9.51±4.39	8.17±4.48
Taxol	19.80±6.74	13.21±5.61	13.27±6.16	10.41±3.62	11.36±4.43	10.91±4.78	9.26±4.29	6.79±3.33
Vinorelbine	19.86±6.46	12.11±4.47	12.28±4.74	11.86±4.72	12.49±3.90	10.58±4.75	9.86±3.99	6.78±3.53

Supplementary Table 10. Average durations of events (in hours) for A549, PC-3 and HaCaT cell lines after the treatment with 1 uM Taxol (T) + 10 uM A-1155463 (A) for A549 and PC-3 cells + 100 uM Z-VAD-FMK (ZVAD).

	Duration of mitotic arrest before MOMP	Duration of mitotic arrest after MOMP	Total duration of mitotic arrest	Duration of post-slippage interphase
**A549 (T+A+ZVAD)**	11.17±3.17	8.27±3.55	19.37±4.2	21.08±5.63
**PC-3 (T+A+ZVAD)**	8.86±4.42	7.54±3.38	16.06±3.84	18.67±9.26
**HaCaT (T+ZVAD)**	7.27±3.9	6.55±3.86	13.51±4.18	24.13±6.96

Supplementary Table 11. Average durations of mitotic arrest (in hours) for HeLa and HaCaT cells after treatment with Barasertib, Taxol + Barasertib simultaneously, Taxol followed by Barasertib (sequentially) and Taxol only.

	Barasertib	Taxol + Barasertib together	Taxol + Barasertib sequentially	Taxol
**HeLa**	1.15±0.31	6.57±3.1	8.73±4.28	12.71±6.04
**HaCaT**	0.93±0.32	6.29±4.45	8.29±3.35	11.31±5.12

Supplementary Table 12. Average durations of mitotic arrest (in hours) for sensitized A549 and PC-3 cells after treatment with Barasertib + A-1155463 (B+A), Taxol + A-1155463 + Barasertib together (T+A+B tog.), Taxol+ A-1155463 + Barasertib sequentially (T+A+B seq.) and Taxol + A-1155463 (T+A).

	B+A	T+A+B tog.	T+A+B seq.	T+A
**A549**	1.13±0.65	3.55±3.23	6.83±3.3	10.77±4.29
**PC-3**	1.38±0.42	3.44±1.37	6.94±3.4	10.22±3.87

Supplementary Table 13. Fates of A549 cells after sequential treatment with mitotic inhibitors (1 μM of Nocodazole, Taxol, or Vinorelbine) followed by 1 μM of A-1155463 (number of cells survived/died)

	Nothing added	Added after 4 h	Added after 20 h
**Nocodazole**	114/1	63/77	78/0
**Taxol**	77/6	16/131	20/47
**Vinorelbine**	57/1	20/113	49/18
